# Micronutrients and Toxic Elements in Soil, Grass, and Nutritional Supplements and in Blood and Meat Products from Beef Cattle Raised in the Southern Amazon, Brazil

**DOI:** 10.1021/acs.jafc.4c12513

**Published:** 2025-06-10

**Authors:** Fernando Luiz Silva, Marcus Henrique Martins e Silva, Ernandes Sobreira Oliveira Júnior, Áurea Regina Alves Ignácio, Marta López-Alonso, Marta Miranda, Veronica Piñeiro, Maria Aparecida Pereira Pierangeli

**Affiliations:** 1 Department of Education, Federal Institute of Mato Grosso, Alta Floresta, MT 78580-000, Brazil; 2 Center for Research on Limnology, Biodiversity, and Ethnoecology, Graduate Program of Environmental Science, University of Mato Grosso State, Cáceres, MT 78200-000, Brazil; 3 Department of Animal Pathology, Faculty of Veterinary, Campus Terra, University of Santiago de Compostela, Lugo 27002, Spain; 4 Department of Anatomy, Animal Production and Veterinary Clinical Sciences, Faculty of Veterinary, Campus Terra, University of Santiago de Compostela, Lugo 27002, Spain; 5 Instrumental Analysis Unit, Network of Infrastructures to Support Research and Technological Development (RIAIDT), Campus Terra, University of Santiago de Compostela, Lugo 27002, Spain; 6 Department of Animal Science, Graduate Program of Environmental Science, University of Mato Grosso State, Pontes e Lacerda, MT 78250-000, Brazil

**Keywords:** farm, livestock, mercury, one health, pasture, selenium

## Abstract

The objectives of this study were to investigate the transfer of micronutrients and toxic elements from the environment to animals and food and to obtain information about trace element levels in pasture-based animal production systems. Samples of soil, grass, nutritional supplements, and blood were obtained from different farms in the southern Amazon, Brazil. In addition, samples of muscle, kidney, and liver were obtained from various markets in the same region. All samples were analyzed to determine the concentrations of several elements: Fe, Mn, Zn, Cu, Se, Co, Mo, Ni, Cr, Pb, As, Cd, and Hg. Low concentrations of these elements were detected in soil and grass samples. Deficient or excessive concentrations of several micronutrients were detected in the nutritional supplements. Marginal Se deficiencies were identified in cattle blood. Meat products had low concentrations of Pb, As, Cd, and Hg and were considered rich sources of micronutrients, particularly of Fe, Zn, Se, and Cr in the muscle, Cu and Mo in the liver, and Se in the kidney. Information about the levels of micronutrients and toxic elements in animal production systems, such as pasture-based systems, can support strategies that promote sustainable farming and environmental, animal, and human health.

## Graphical abstract

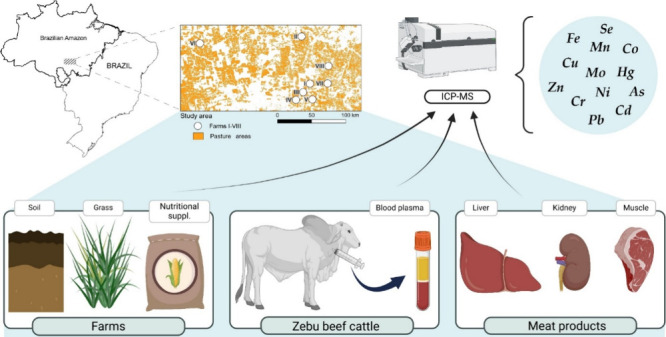

## 1 Introduction

The production of animal-source foods is facing many global challenges related to climate change, land use efficiency, and food security, among others. Animal grazing systems are important in this context, as they occupy approximately one-third of the earth’s surface.^1,2^ Pasture-based animal production systems supply high-quality protein, with a low level of competition with human food, and they are an important source of income in many regions.^3–5^ Pasture-based systems can also be established in areas that are not suitable for agriculture or as part of integrated systems, thus favoring soil health, control of invasive plants, and animal welfare.^6–8^ However, animal grazing systems can also have negative impacts, such as increased deforestation, low productivity rates, and environmental degradation.^1,2^ Understanding how nutrient pathways interact can facilitate application of the One Health concept (which aims to optimize environmental, animal, and human health) in strategies aimed at improving livestock rearing conditions, productivity, food quality, and sustainable development.^9^

The challenges associated with supplying healthy food and implementing sustainable production methods are subject to a broad debate. In this context, consumption of animal-source foods is often recommended to ensure an adequate supply of nutrients such as proteins, vitamins, and minerals.^10^ Meat and offal are important sources of many micronutrients, including Fe, Zn, Cu, and Se, in the human diet.^11,12^ Micronutrients play essential roles in metabolism by participating in enzyme reactions, including those involved in helping the immune system to fight infections and prevent diseases.^13^ Selenium deficiency, for example, has been linked to a number of human diseases, including diabetes, cardiovascular diseases, immune system disorders, and cancer.^14^ On the other hand, contamination of food by potentially toxic elements is of growing concern.^15^ Unlike micronutrients, toxic elements such as Pb, Cd, As, and Hg do not have a known function in metabolism and can threaten human health even when present at very low concentrations.^16^ The presence in food of elements known to be toxic should therefore be avoided, and prevention strategies must be adopted to guarantee food safety.^17^

Monitoring the levels of micronutrients and toxic elements is also important in animal nutrition and environmental quality assessment. Establishing diets that meet nutritional requirements is a challenging aspect of animal nutrition.^18^ Although the dietary requirements of cattle are well established for most micronutrients,^19^ wide margins exceeding the nutritional requirements for many micronutrients are permitted in livestock to ensure supply of minimum levels.^20^ This leads to the risk of intake of excess nutrients, for example, Cu or Se, for which the limits between requirement and toxicity are very narrow.^21–23^ Furthermore, there is an ever-present risk of residues of toxic elements being present above the permitted levels in livestock diets,^24^ as well as the continuous excretion of micronutrients or toxic elements through feces and urine, which can lead to environmental contamination.^25^

In the Brazilian Amazon, about 55 million hectares of pasture are used for cattle ranching, more than half of which are moderately or severe degraded.^26^ In this context, the pasture does not usually meet bovine nutritional requirements,^27^ and some deficiencies can be aggravated. There is also a risk of trace elements being released to the environment due to the use of fertilizers, herbicides, and insecticides or due to excessive mineral supplementation in cattle.^20,21^ Fires and mining (mainly artisanal gold mining) also threaten the environment through the release of Hg into the atmosphere followed by uptake by pasture grasses.^28,29^ These sources of contamination can result in the accumulation of trace elements, which can eventually reach toxic levels for microorganisms, plants, animals, and foods.^15^

Studies concerning the transfer of micronutrients and toxic elements between the soil, plants, and animals in pasture systems are scarce. Given the importance of the topic, the present study determined the concentrations of Fe, Mn, Cu, Zn, Se, Co, Mo, Ni, Cr, Pb, As, Cd, and Hg in samples of soil, grass, cattle nutritional supplements, and cattle blood in pastures in the southern Brazilian Amazon. The concentrations of these elements were also determined in samples of meat products (muscle, kidney, and liver) obtained from markets in the same region. In addition to determining the concentrations of these elements, the study objectives were as follows: (a) to investigate the transfer of the elements from soil to plants to animals; (b) to evaluate cattle diets in regard to element requirements and intake limits; (c) to evaluate the blood nutritional status of pasture-reared cattle; (d) to evaluate whether cattle-derived food products meet the element requirements and recommended limits for human consumption; and (e) to obtain more information about pasture-based animal production systems.

## 2 Material and Methods

### 2.1 Study Area and General Descriptions

Sampling was carried out on eight extensive pasture-based beef farms (I–VIII) located in the southern Brazilian Amazon (Figure 1) between October 2021 and March 2022 (rainy season). According to the Köppen classification,^30^ the climate in the region is Am, with average temperatures between 20 and 35 °C and rainfall between 2000 and 3000 mm year^–1^. The study region is located in the “Arc of deforestation” in the Amazon,^31^ considered one of the most important agricultural areas in the region. Three of the farms (II, III, and V) were located within 5 km of historical artisanal gold mining areas.

**Figure 1:**
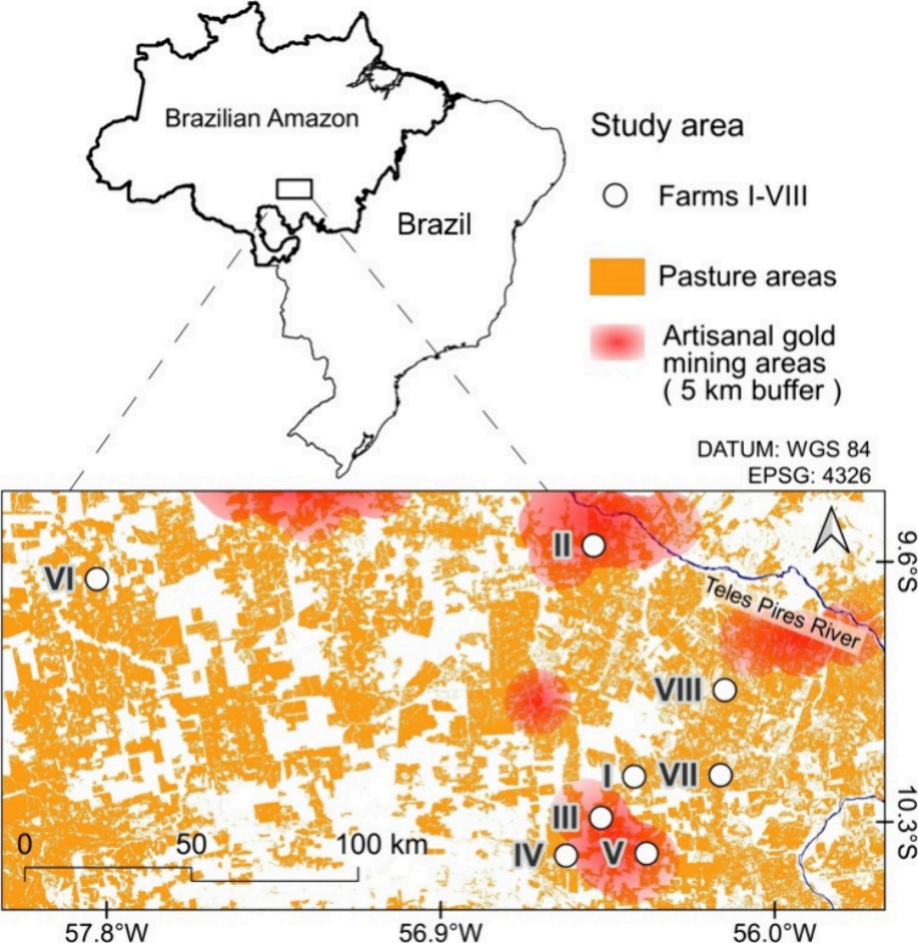
Location of the study area and farms. The geographic features of the pasture areas were obtained from MapBiomas.^26^ Locations of artisanal gold mining were obtained from the *Serviço Geológico do Brasil.* ^32^

Soil, grass, and nutritional supplements provided to the herds were sampled on all farms. In addition, a semistructured questionnaire was administered to the farmers to obtain technical and productive information and information about the herd management history of each farm (University of the State of Mato Grosso, Brazil, Research Ethics Committee: 44321521.5.0000.5166). General information about each farm and the macronutrient fertility conditions has already been reported.^33^

Blood samples were obtained from female Nellore (zebu beef cattle) during the growing or finishing phase from five farms (I, II, III, IV, and VI) (University of the State of Mato Grosso, Brazil, Ethics Commission in the Use of Animals: Process 001/2021). On farm VI, two herds were sampled, so blood samples from a total of six herds were obtained in this study. Blood samples were not able to be obtained from cattle on farms V, VII, and VIII due to difficulties in safely transporting the animals to the handling site or due to the lack of adequate facilities for the safe handling of the animals. Some general information about each herd is provided in Table S1.

Samples of meat products (muscle, kidney, and liver) were obtained from markets in the city of Alta Floresta, State of Mato Grosso, Brazil, one of the main centers of commercial beef production in the region.

### 2.2 Sampling Procedure

Soil samples were obtained from each farm. Samples were collected from the upper 0.0–0.2 m soil layer at sampling points separated by at least 50 m. Three individual subsamples were collected (at least 10 m apart) at each sampling point and combined to produce a composite soil sample. A total of 24 composite soil samples, of approximate weight of 500 g, were thus obtained. The samples were sent to the laboratory, where they were air-dried and processed by passing through a 2 mm sieve to yield the fine earth fraction. Each sample was then macerated until it passed through a 150 μm sieve and was then stored until chemical analysis.

Grass was also sampled in each pasture where the cattle were grazing. Three sampling points each measuring 0.8 × 0.8 m were selected at random, and the grass was cut to a height of 5 cm above the soil. In the laboratory, the grass leaves were separated from the sheath, dried at 60 °C for 72 h, ground to a 1 mm thickness, and stored under a vacuum until chemical analysis.

Samples of nutritional supplements (500 g) were collected on each farm. Information about the supplements and instructions for use were obtained from the product labels. The samples were dried at 105 °C for 72 h in the laboratory to determine the moisture content, and they were then ground to 1 mm. The samples were stored in vacuum packages until chemical analysis.

Samples of cattle nutritional supplements were classified as follows: (a) corn silage, feed with a crude fiber content of more than 18% of dry matter (DM) and including a mineral premix, which was supplied in variable quantities to grazing cattle; (b) protein/energy supplemented feed, with a crude fiber content of less than 18% of DM and typically containing ground corn grain, soy bran, and mineral premix, was consumed at an estimated rate of between 500 and 2700 g per animal unit of 450 kg body weight (AU) per day; (c) mineral block, provided in block form and mainly composed of sugar cane molasses, was consumed at an estimated rate of between 100 and 450 g/AU per day; (d) mineral powder, mineral supplement provided in powder form mainly composed of a mixture of ground rock and sodium chloride (NaCl), was consumed at an estimated rate of between 80 and 135 g/AU per day; and (e) mineral premix, typically included in the corn silage or protein/energy supplements and not consumed directly by cattle. More information on each nutritional supplement sampled is provided in Table S2.

Four Nellore cows were selected at random from each herd for blood sampling. The blood samples were collected by jugular venipuncture in a 10 mL tube with sodium heparin and centrifuged for 20 min (10G), yielding approximately 4 mL of blood plasma. The blood plasma samples were refrigerated (4 °C) and transported to the laboratory where they were stored at −20 °C. Once blood samples were obtained from all herds, the stored blood plasma samples were thawed, transferred in 0.5 mL aliquots to 2 mL tubes, and subsequently dried for 72 h at 60 °C. The tubes with the dried samples were stored by vacuum packing and held under refrigeration (4 °C) until chemical analysis.

Samples of muscle (*longissimus dorsi*) and offal (liver and kidney) were collected between April and May 2022 from eight commercial establishments. Four samples of the muscle, liver, and kidney, each weighing approximately 300 g, were obtained weekly from different markets until a total of 32 samples of each tissue were obtained. Immediately after collection, the samples were refrigerated (4 °C) and transported to the laboratory for analysis. All fat, connective tissue, and bones were removed from the samples. About 100 g of each meat product sample was dried at 105 °C to determine the moisture content. The samples were then ground and then stored under vacuum and refrigeration (4 °C) until chemical analysis.

### 2.3 Chemical Analysis

All samples were analyzed by inductively coupled plasma mass spectrometry (ICP-MS) to determine the concentrations of Fe, Zn, Cu, Mn, Se, Co, Mo, Ni, Cr, Cd, Pb, As, and Hg. The sample introduction system consisted of an autosampler, a double-stage spray chamber with a Peltier system (Agilent Technologies, Tokyo, Japan), a concentric glass nebulizer (MicroMist, low flow, Glass Expansion, West Melbourne, Australia), and a quartz torch (Agilent Technologies, Tokyo, Japan). The element concentrations were quantified with dedicated software (Agilent ICP-MS MassHunter 5.1, Version D.01.01, Agilent Technologies, Tokyo, Japan). The following operating parameters were applied: plasma gas flow of 15 L/min; nebulizer gas flow of 1.1 L/min; sampling depth of 8; sampling speed of 0.1 rpm; plasma RF power of 1550 W; and a spray chamber temperature maintained at 2 °C. Helium (He) gas was used for interference correction. In all cases, a linear fit was obtained for the blank- corrected data, and the correlation coefficient was >0.999.

The samples of soil, grass, nutritional supplements, and meat products were macerated and dried at 105 °C. Aliquots of about 0.3 g (±0.003) of each type of sample were digested by adding 3 mL of HNO_3_ (69%) and 1 mL of ultrapure water and subjected to a microwave-assisted acid digestion protocol (ultraWAVE Milestone, Sorisole, Italy) at 240 °C and 40–120 bar for 1 h prior to analysis. The digested samples were then each made up to 50 mL with ultrapure water, and the element contents were determined by ICP-MS. For soils, this method provides the pseudototal concentration, as the crystalline matrix is not completely decomposed during digestion. However, the method is widely used in the context of environmental assessment, as it indicates the bioavailable quantity in the long term of the element in the soil.^34^

To dissolve the dehydrated blood plasma samples, 0.5 mL of ultrapure water (Milli-Q) and 0.5 mL of HNO_3_ (69%) were added. The mixture was then transferred to 5 mL tubes, to which 0.3 mL of H_2_O_2_ was added, and centrifuged for 5 min. The solution was held in an oven at 60 °C for 12 h to allow the complete dissolution of the samples. The volume was then made up to 4 mL with ultrapure water, and the element contents were also determined by ICP-MS.

For the analytical control of soil, grass, nutritional supplements, and meat products, certified reference samples were included in each sample batch. For analytical control of the blood plasma, a standard serum sample, enriched with 5, 10, and 50 μg/L of the trace elements of interest, was included. Analytical blanks were also included in each batch, and the data obtained were used to calculate the limit of detection (LOD), according to the following equation: ["white" sample mean + (3 × standard deviation)] (Table S3). The concentrations of elements in the reference samples and the recovery rates of the method are listed in Table 1 and 2. The results obtained for the certified reference materials and the spiked samples showed satisfactory recovery levels, and the accuracy of the determinations was therefore considered acceptable.

**Table 1:** Analytical Quality Control Carried Out during the Analysis of Trace Elements in Soil, Grass, Nutritional Supplements, and Meat Products^a^

	CRM soil^b^			CRM apple leaves^c^			CRM wheat^d^			CRM bovine liver^e^			CRM fish muscle^f^		
Element (mg/kg)	cert	meas	rec%	cert	meas	rec%	cert	meas	rec%	cert	meas	rec%	cert	meas	rec%
Fe	4,280.0	4,315.6	100.8	83.0	67.0	80.7	18.5	12.3	66.3		203.7			11.3	
Mn	335.0	406.9	121.5	54.0	44.5	82.5	5.4	4.2	78.0	11.1	11.1	100.4	0.4	0.4	111.2
Zn	770.0	742.0	96.4	12.5	10.5	83.9	11.6	9.0	77.7	138.6	154.5	111.5	16.0	20.0	124.7
Cu	78.9	76.7	97.2	5.6	4.6	82.2	2.7	2.4	90.2	277.0	297.9	107.5	1.7	1.9	116.8
Mo	57.8	52.5	90.7	0.1	0.1	86.2	0.5	0.4	86.3		3.8			0.01	
Co	60.1	59.0	98.2	0.1	0.1	84.4		0.0			0.2			0.01	
Se	42.4	33.5	78.9	0.05	0.07	149.2	0.05	0.09	172.9	1.7	1.3	79.4	1.3	1.2	89.8
Ni	143.0	151.1	105.7	0.9	0.7	80.5		0.02			0.01			0.02	
Cr	179.0	217.2	121.3	0.3	0.2	78.3	0.01	0.01	128.2		0.04			0.02	
Pb	145.0	162.6	112.1	0.5	0.4	82.2		0.02		0.2	0.1	83.2		0.004	
As	123.0	124.7	101.4	0.04	0.07	171.9	0.03	0.02	55.0	0.03	0.03	79.8	12.7	13.4	105.2
Cd	224.0	227.5	101.6	0.01	0.01	102.2	0.02	0.01	77.1	0.5	0.5	89.3	0.01	0.01	73.5
Hg	4.6	4.9	106.3	0.04	0.04	99.8					0.004		0.6	0.6	97.2

a Abbreviations: CRM = certified reference material; cert = certified; meas = measured; rec% = recovery in percentage.

b MR SQC001 soil.

c MR NIST 1515 apple leaves.

d MR GBW10011 wheat.

e MR 1-185 bovine liver.

f MR 4-BB422 fish muscle.

**Table 2:** Analytical Quality Control Carried Out during the Analysis of Trace Elements in Blood Plasma

		level added (μg/L)			measured (μg/L)			recovery (%)		
element	blood serum	5	10	50	5	10	50	5	10	50
Fe	304.29	309.29	314.29	354.29	317.61	324.97	374.10	103	103	106
Mn	0.46	5.46	10.46	50.46	4.56	10.46	52.43	84	100	104
Zn	153.10	158.10	163.10	203.10	162.10	168.99	217.42	103	104	107
Cu	117.56	122.56	127.56	167.56	123.74	130.61	173.94	101	102	104
Mo	7.38	12.38	17.38	57.38	10.96	15.69	49.81	89	90	87
Co	0.10	5.10	10.10	50.10	3.95	9.32	48.16	77	92	96
Se	13.34	18.34	23.34	63.34	17.63	26.74	64.26	96	115	101
Ni	0.09	5.09	10.09	50.09	4.00	9.52	48.82	79	94	97
Cr	0.25	5.25	10.25	50.25	4.30	9.84	50.77	82	96	101
Pb	0.16	5.16	10.16	50.16	3.72	8.49	41.80	72	84	83
As	1.02				1.06	1.02	1.02			
Cd	0.01	5.01	10.01	50.01	3.91	9.31	47.39	78	93	95
Hg	0.48				0.29	0.24	0.17			

All samples, except blood plasma, were also analyzed using a Direct Mercury Analyzer (Milestone DMA-80) to determine the Hg levels. The available contents of Fe, Mn, Cu, and Zn in soil were also analyzed by flame atomic absorption spectrometry (F-AAS) after extraction with Melich-1 solution.^35^

### 2.4 Statistical Analysis

Descriptive statistics of the concentration of trace elements (Fe, Mn, Zn, Cu, Se, Co, Mo, Ni, Cr, Cd, Pb, As, and Hg) in soil and grass (eight farms), nutritional supplements (corn silage, protein/energy, mineral powder, or mineral premix), blood plasma (six herds), and meat products (muscle, kidney, and liver) were obtained. Pearson’s correlation analysis was used to evaluate the possible transfer of trace elements between soil and grass. The elements for which the correlation ≥ 0.90 and *p* value ≤ 0.001 were subjected to regression analysis.

The total daily DM consumption by cattle was assumed to be 9.5 kg, considering zebu beef cattle in a pasture system with body weight equivalent to 1 AU (animal unit of 450 kg). The amount of each nutritional supplement assumed to be consumed daily was equivalent to the recommendation presented on the label of each sampled product calculated from the DM weight (Table S2). The amount of grass consumed daily was estimated as the difference between the total daily consumption (9.5 kg) and the daily consumption of each nutritional supplement.

The intake of trace elements by cattle was estimated considering the combined consumption of nutritional supplements and grass using eq 1:

$$\mathrm{DI}=\frac{\left(S\times \mathrm{ES})+(G\times \mathrm{EG}\right)}{9.5}$$ 1

where DI is the daily intake of the element per AU in mg/kg DM; *S* is the daily consumption of the nutritional supplement per AU in kg DM; ES is the element content in the nutritional supplement in mg/kg DM; *G* is the daily consumption of grass per AU in kg DM; EG is the element content in the grass in mg/kg DM; and 9.5 is the daily consumption of feed per AU in kg.

The intake of trace elements from the consumption of meat products (muscle, kidney, and liver) was analyzed in various different target groups (sex, age, and body weight), as follows: men, 30 years old, 80 kg; women, 30 years old, 65 kg; boys, 16 years old, 58 kg; girls, 16 years old, 53 kg; and children, 6 years old, 20 kg.^36–38^ A fixed monthly consumption of 1000 g of muscle, 100 g of kidney, and 100 g of liver was assumed for all groups.^39^ The element content used for each food was the range between the 10th and 90th percentile of the descriptive results of the muscle, kidney, and liver of cattle.

The relative monthly intake (RMI) obtained from consumption of the muscle, kidney, and liver based on the reference daily intake adjusted to 30 days (RDI_30d_) or tolerable upper level adjusted to 30 days (UL_30d_) was obtained using eq 2:

$$\mathrm{RMI}=\frac{\mathrm{FC}\times \mathrm{EF}}{\mathrm{IRL}}\times 100$$ 2

where RMI is the relative monthly intake based on the RDI_30d_ or UL_30d_ expressed as a percentage (%); FC is the monthly amount of meat product consumed in kg (1.0 to muscle; 0.1 to kidney; 0.1 to liver); EF is the element content in meat product in mg/kg; IRL is the RDI_30d_ or UL_30d_ in mg/kg; and 100 is the adjustment for percentage.

Dietary reference values for human intake were obtained from the Food and Agriculture Organization of the United Nations/World Health Organization (FAO/WHO), European Food Safety Authority (EFSA), and Institute of Medicine (IOM). The RDI_30d_ and UL_30d_ values for the categories used in this study are provided in Table S4.

## 3 Results

### 3.1 Trace Elements in the Soil, Grass, and Nutritional Supplements

The descriptive statistics of the pseudototal contents of elements in the soil are listed in Figure 2. The concentrations of Mn, Cr, Pb, As, Cd, and Hg were in the typical range for world soils, while the concentrations of Fe (farms IV, VII, and VIII) were higher than usual. Zinc concentrations were generally below 30 mg/kg (i.e., very low). The concentrations of Co, Mo, and Ni varied between typical and very low. Copper and Se were predominantly present at very low concentrations, although within levels typical of world soils. The concentrations of the element varied widely among farms (Figure 2). Notably, the Se concentrations on farm II were high, as were those of Mo on farms IV and V and those of Hg on farms II and VI.

**Figure 2:**
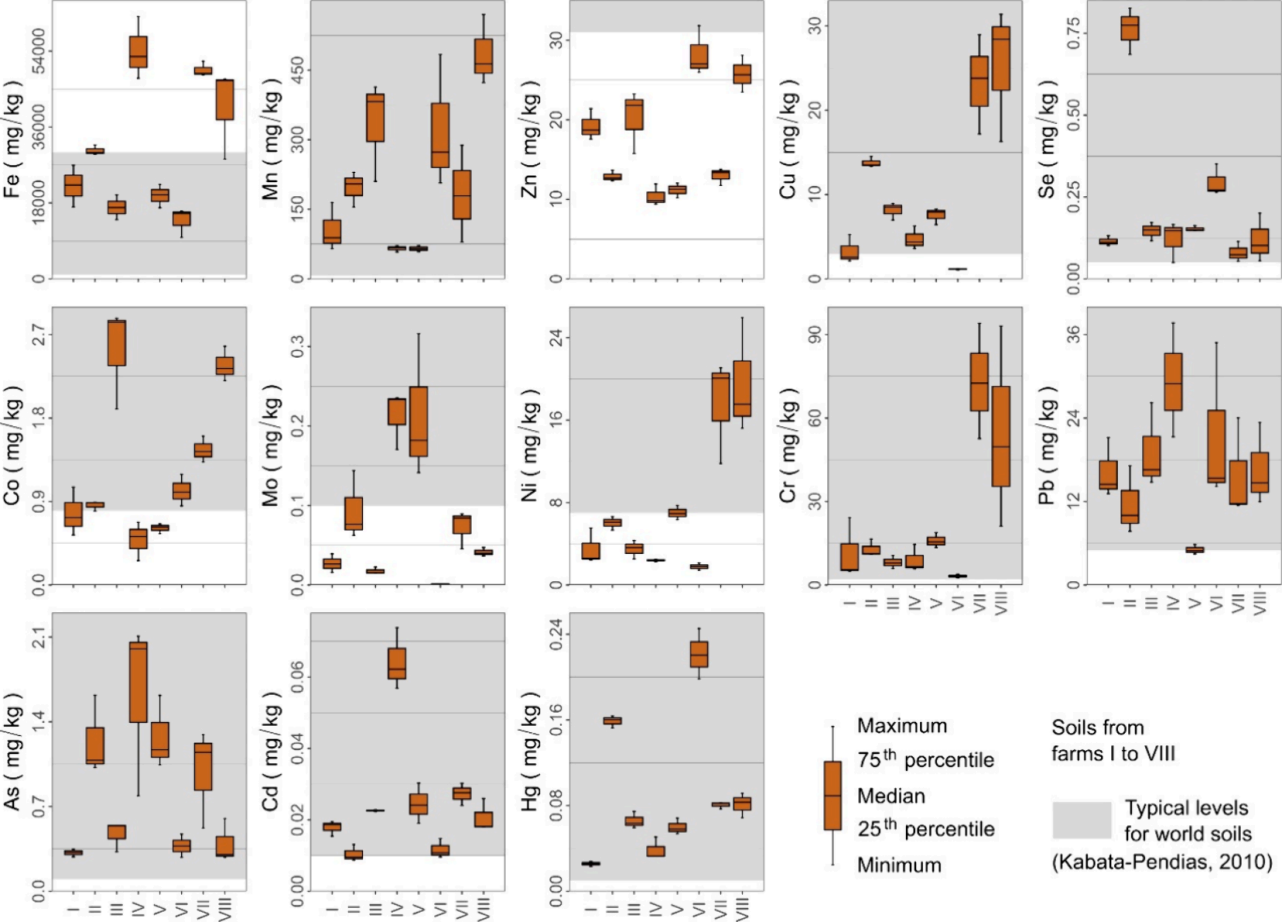
Descriptive statistics of pseudototal contents of trace elements in the soil of pastures from farms I–VIII located in the southern region of the Amazon. Typical levels for world soil from Kabata-Pendias.^40^

The mean concentrations (in mg/kg) (and standard deviations) of available Fe, Mn, Zn, and Cu extracted by Mehlich-1 solution were 81.71 ± 13.57, 21.53 ± 13.11, 0.58 ± 0.21, and 0.57 ± 0.36, respectively (Figure S1). The proportions of these available concentrations in relation to the pseudototal concentrations (Figure 2) were equivalent to 0.3 ± 0.2% for Fe, 10.8 ± 3.0% for Mn, 3.6 ± 1.3% for Zn, and 8.1 ± 5.1% for Cu. Among these elements, considering the soil fertility interpretation tables for the central region of Brazil,^41^ only Fe was present at adequate levels for pastures on all farms (Figure S1; >31 mg/kg), while the levels of Zn and Cu were deficient (<1.6 and 1.3 mg/kg, respectively). Levels of manganese were deficient or intermediate on three of the farms (farms IV, V, and VII; <9.0 mg/kg).

The element contents of the grass are shown in Figure 3. High Mn concentrations were detected in all of the pastures, with values above 60 mg/kg, considered adequate to meet the needs of beef cattle.^19,42^ The median concentrations of Fe, Zn, Cu, Se, and Co were predominantly deficient according to the requirements for zebu beef cattle.^42^ The other elements were also present at very low concentrations in the grass samples. Although Mo, Ni, and Cr are considered essential micronutrients for cattle, requirement levels have not been established by the NASEM,^19^ as deficiencies are not observed under natural conditions. However, Mo and Cr supplementation in zebu beef cattle has been recommended^42^ to provide maintenance and weight gain, and these elements were predominantly deficient in these grasses. Lead, As, Cd, and Hg are considered toxic elements and were present at levels below the respective limits tolerated in beef cattle feed according to NASEM.^19^

**Figure 3:**
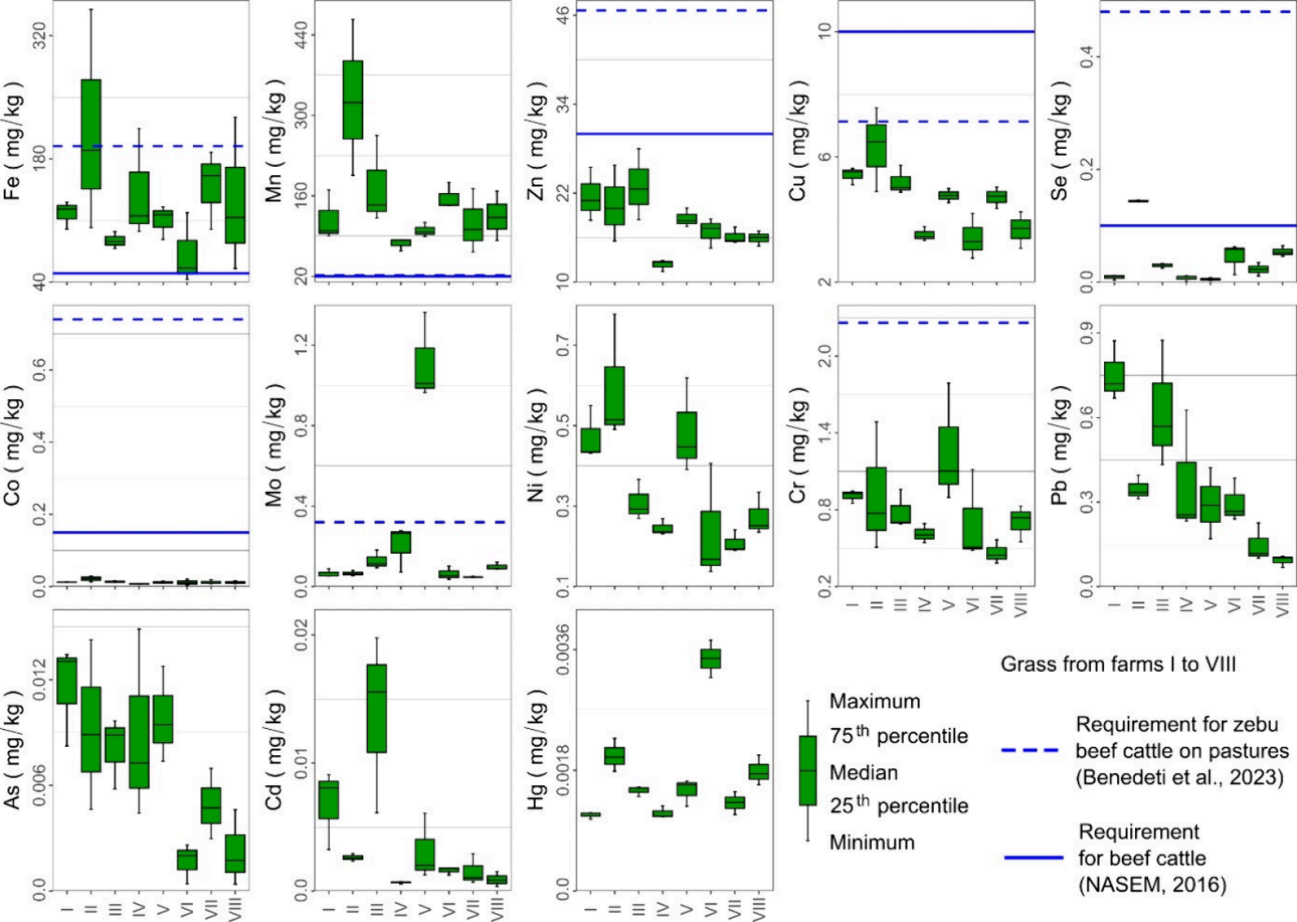
Descriptive statistics of trace elements in the grass (in dry matter) from pastures on farms I–VIII located in the southern region of the Amazon. Hg data in the grass were obtained using a Direct Mercury Analyzer, as it was not possible to detect Hg in some samples using ICP-MS.

The highest concentrations of Se, Mo, and Hg in the grass samples corresponded to farms II, V, and VIII, respectively. The highest concentrations of these elements were also found in the soils on these farms. However, strong correlations between soil and grass were only observed for Se and Hg (Pearson’s correlation ≥ 0.9; *p* ≤ 0.001; Figure S2). Linear regressions between soil and grass for Hg or Se are shown in Figure S3. The coefficients of determination were 0.88 for Hg (*p* < 0.001) and 0.83 for Se (*p* < 0.001).

The element contents in the different categories of nutritional supplements varied greatly (Figure 4). In general, the levels were ranked in the following order: premix > mineral powder > mineral block > protein/energy > corn silage. However, high Fe, Ni, Pb, and Cd concentrations were detected in most of the samples of mineral powder, and high Cr and As concentrations were detected in one sample of mineral block.

**Figure 4:**
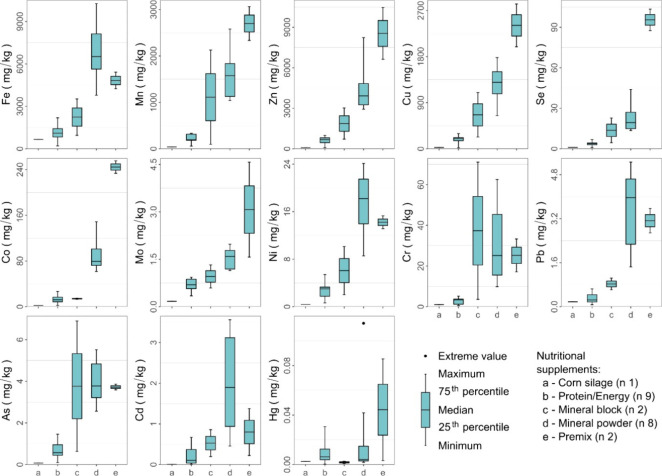
Descriptive statistics of trace elements in bovine nutritional supplements (in dry matter) offered to cattle herds reared on farms located in the southern region of the Amazon. *n* = number of samples obtained from each nutritional supplement.

### 3.2 Estimated Intake of Elements by Zebu Beef Cattle

The estimated consumption of each nutritional supplement and grass and the estimated intake of the elements are provided in the Supporting Information (Table S5–S7). A few examples where the concentrations of elements in the diet were below, above, or marginal to requirements and limits are shown in Figure 5. In this figure, the observed variation between points on the *x* axis represents the concentrations measured in the grass samples. Thus, the combination of each nutritional supplement in these pastures provides varying levels of each element to the animal diet. The vertical lines on the *x* axes indicate whether the diets meet the appropriate requirements for cattle. Estimated intakes of Fe and Cu in two diets were above the tolerated limits for cattle; however, for Fe, this occurred only when the nutritional supplements were combined with grass containing a high level of this element. Considering the requirements for zebu beef cattle,^42^ which are generally higher than those of the NASEM,^19^ Cu, Zn, Se, and Co were deficient or marginally deficient in some diets depending on the element content in the grass (Figure 5).

**Figure 5:**
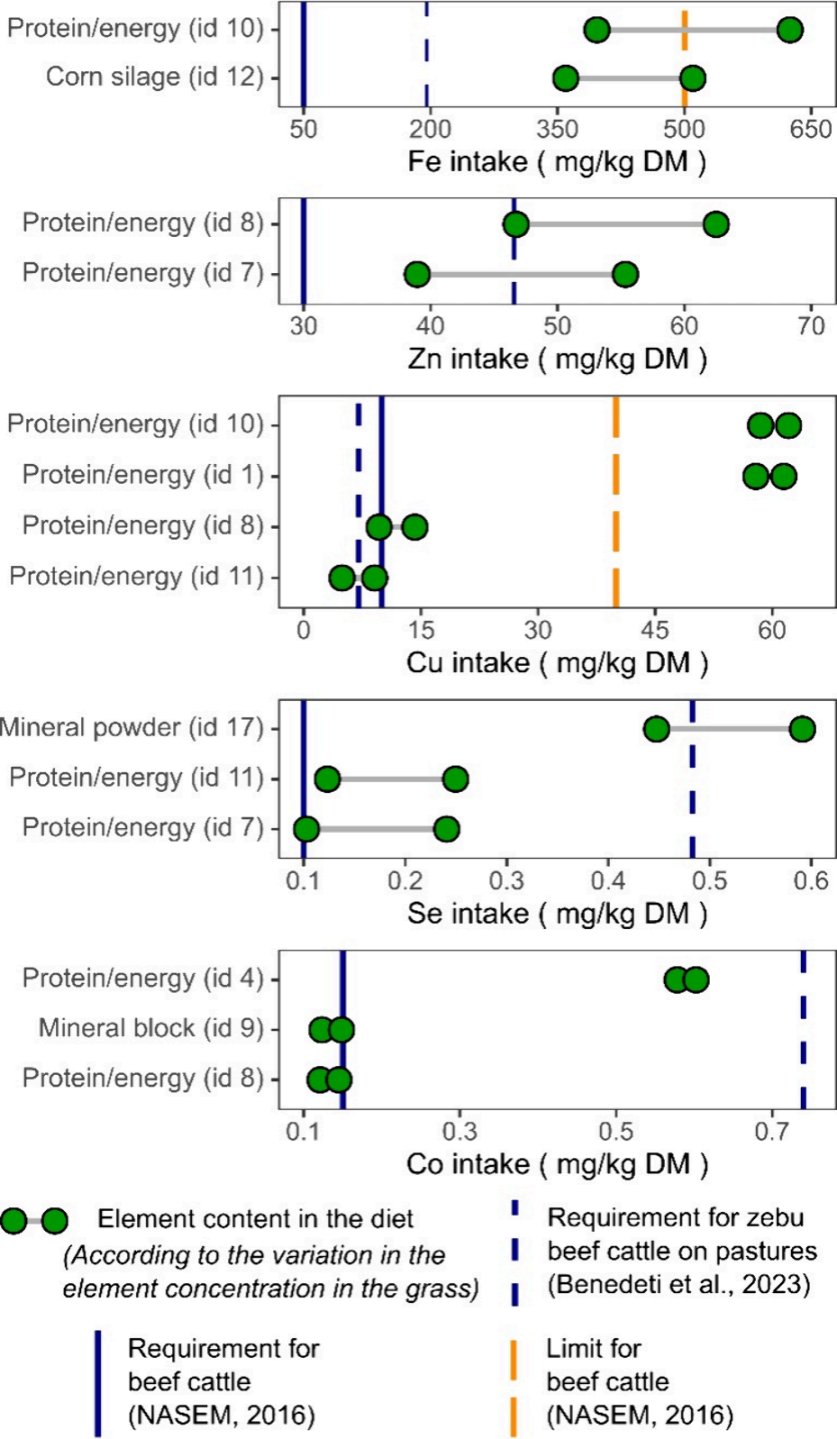
Estimate of trace element intake by cattle on pasture through consumption of grass and nutritional supplements in the southern region of the Amazon. The estimate considers the following range between the minimum and maximum levels of each element in the grass: (in mg/kg DM) 43.07–349.83 for Fe, 11.43–27.99 for Zn, and 2.77–7.57 for Cu; (in μg/kg DM) 0.79–145.94 for Se and 4.19–29.51 for Co. Estimated intakes for all elements and combinations of grass and nutritional supplements are provided in Tables S5–S7.

### 3.3 Elements in the Blood Plasma of Zebu Beef Cattle and in the Meat Products

The element levels in blood plasma are shown in Figure 6. Concentrations of Fe, Mn, Zn, Cu, and Co were within the normal range (or slightly higher) for beef cattle. Concentrations were only within the normal range in herd II for Se and herd IV for Mo, and they were lower in the other herds. The herds with the highest Se and Mo levels in the blood plasma were those with the highest Se and Mo levels in the soil and grass. The other elements (Ni, Cr, Pb, As, Cd, and Hg) were within the range reported in the literature for healthy beef cattle.

**Figure 6:**
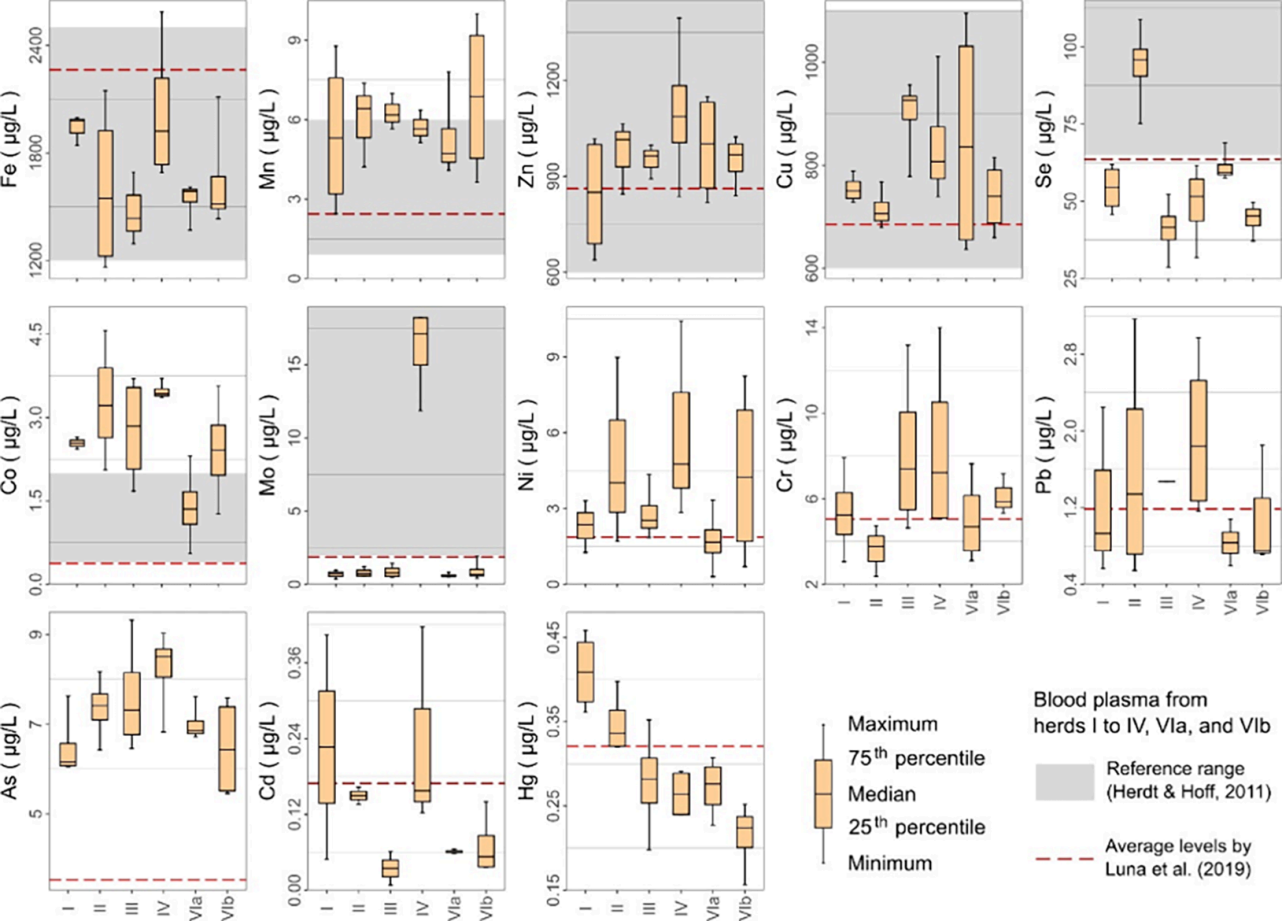
Descriptive statistics of trace elements in the blood plasma of herds I to IV, VIa, and VIb from farms located in the southern region of the Amazon. No reference ranges were available for Ni, Cr, Pb, As, Cd, and Hg.^43^

The element concentrations in the muscle, kidney, and liver samples obtained from markets in the southern region of the Amazon are shown in Figure 7. The element concentrations were lower in the muscle than in the kidney and liver, except those of Zn and Cr, which were similar to those detected in the liver. The Se, As, Cd, and Hg levels were highest in the kidney, while those of Fe, Mn, Cu, Co, Mo, and Ni were highest in the liver. Concentrations of Pb were similar in the liver and kidney, although they were more variable in the liver. No meat products contained elements at concentrations higher than the permitted limits for human consumption.^44–47^

**Figure 7:**
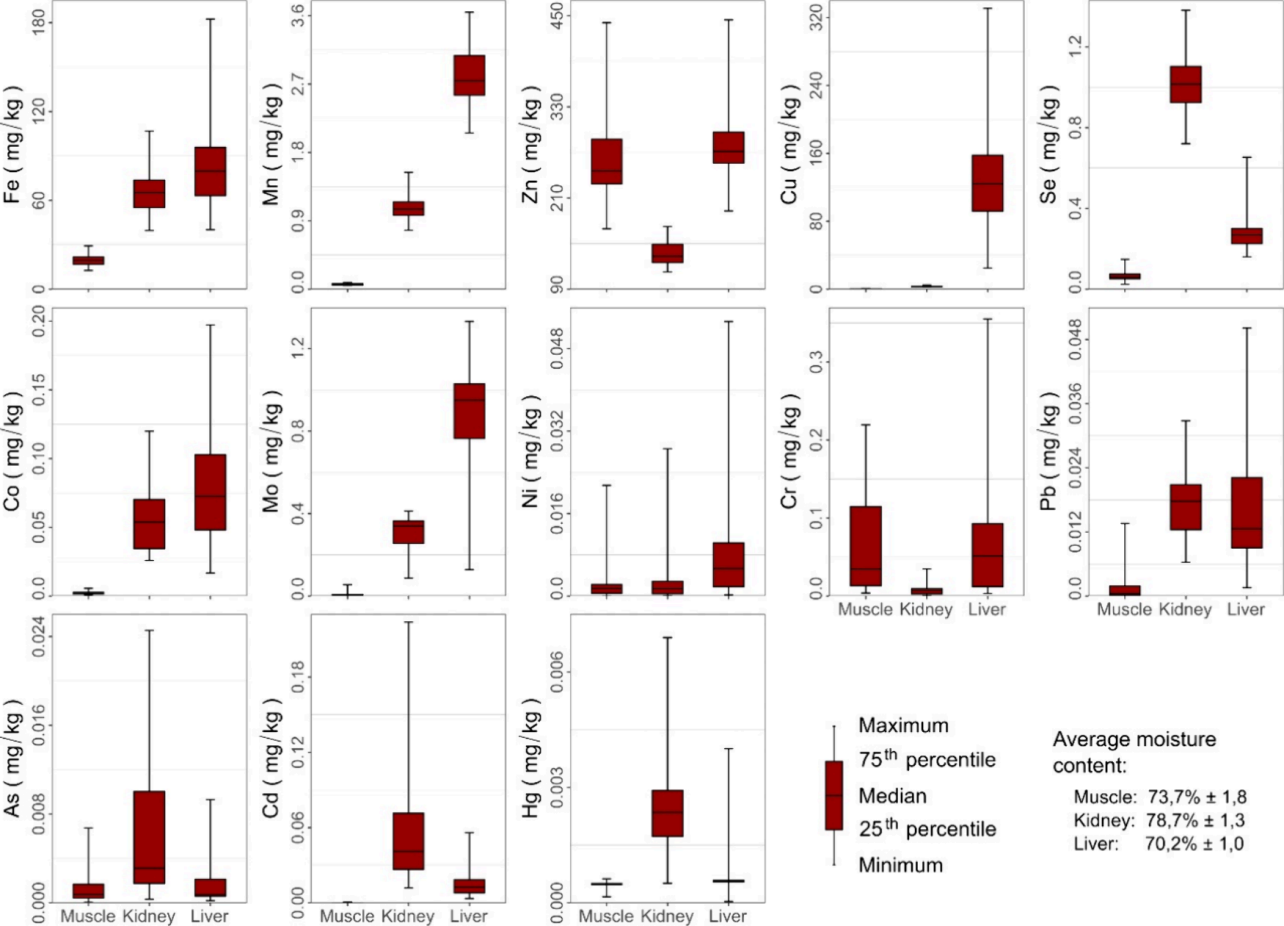
Descriptive statistics of trace elements in the muscle, kidney, and liver (wet weight) obtained in markets located in the southern region of the Amazon. The minimum, 10th, 25th, 50th, 75th, 90th, and maximum percentiles of elements in meat products are shown in Tables S7–S9.

### 3.4 Estimated Intake of Elements by Humans from the Consumption of Meat Products

Estimated intakes of micronutrients based on RDI_30d_ from the consumption of meat products (muscle, kidney, and liver) for some groups over a monthly period are shown in Figure 8. The observed variation between the points on the *x* axis represents the concentration of each element in the meat product samples (range between the 10th and 90th percentiles). Thus, the relative contribution to meeting the RDI_30d_ is greater when there is a high concentration of the element in the food. The consumption of 1000 g of muscle represented a very high contribution in relation to the Zn intake, reaching RDI_30d_ of more than 200% for children and more than 50% for the other groups. Furthermore, this also represented an important contribution in relation to the intake of Fe, Se, and Cr, especially the 90th percentile of the respective levels found in the muscle (>4, >7, and >15%, respectively). The kidney provided useful levels of Se (>5% of RDI_30d_), while the liver provided useful levels of Cu (>20% of RDI_30d_), despite the estimate assuming a monthly consumption of only 100 g of the kidney or liver.

**Figure 8:**
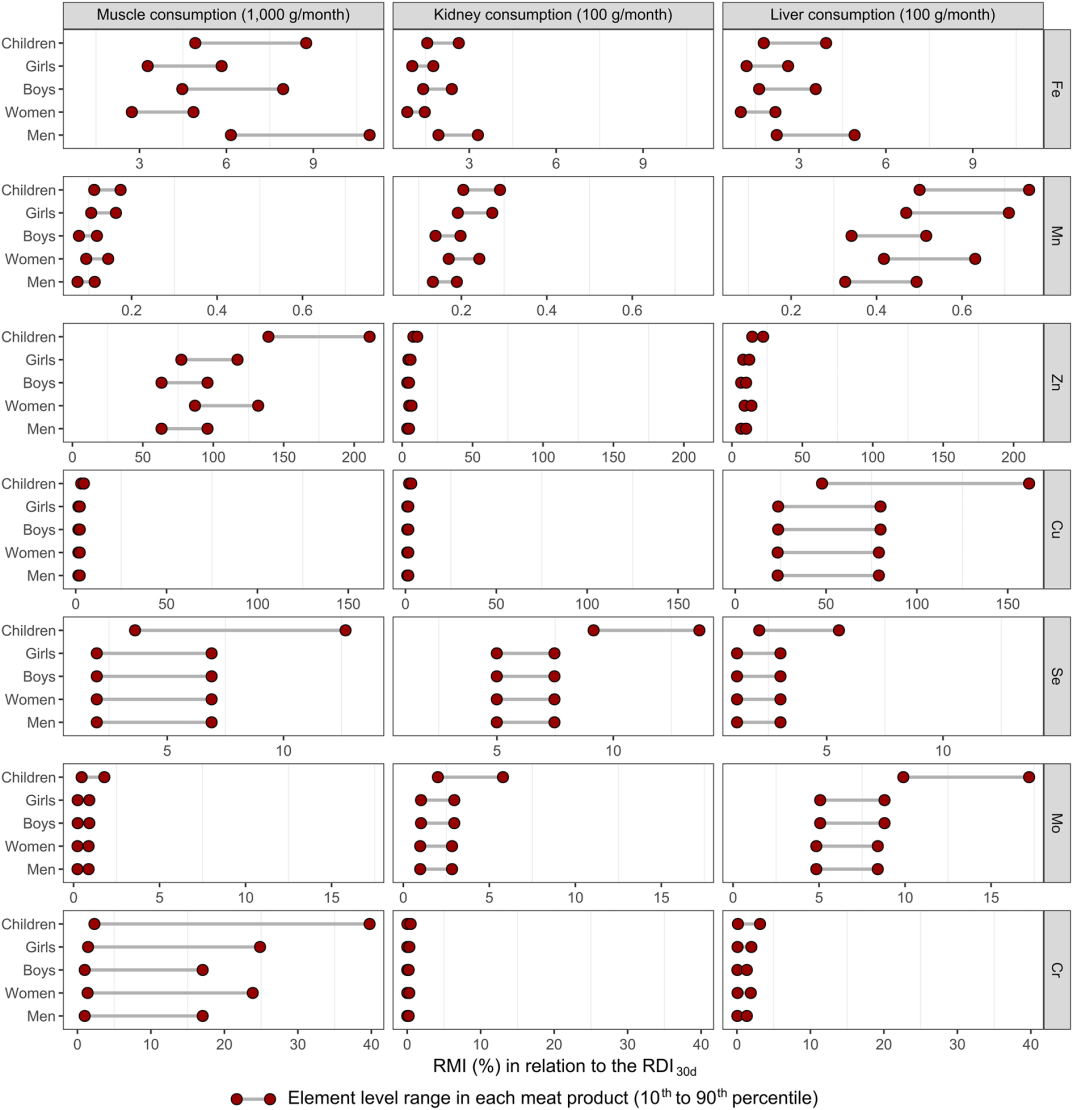
Estimate of relative monthly intake (RMI) to the reference daily intake adjusted to 30 days (RDI_30d_) for human categories by consumption of the muscle, kidney, and liver of cattle obtained in markets located in the southern region of the Amazon. The following ages and body weights were assumed for the different human categories: 6 years and 20 kg for children; 16 years and 53 kg for girls; 16 years and 58 kg for boys; 30 years and 65 kg for women; and 30 years and 80 kg for men. Element contents in the meat products in this figure were the range between the 10th and 90th percentiles of the descriptive results of the muscle, kidney, and liver (Tables S8–S10).

Intake levels based on UL_30d_ estimated for Ni, Pb, As, Cd, and Hg are shown in Figure 9. The estimated intake of Ni, As, and Hg, considering the amounts of food consumed, reached UL_30d_ of less than 0.25% for all groups. Lead and Cd had the highest UL_30d_ for children, reaching about 2% of Pb from the muscle, kidney, and liver and above than 5% of Cd from the kidney.

**Figure 9:**
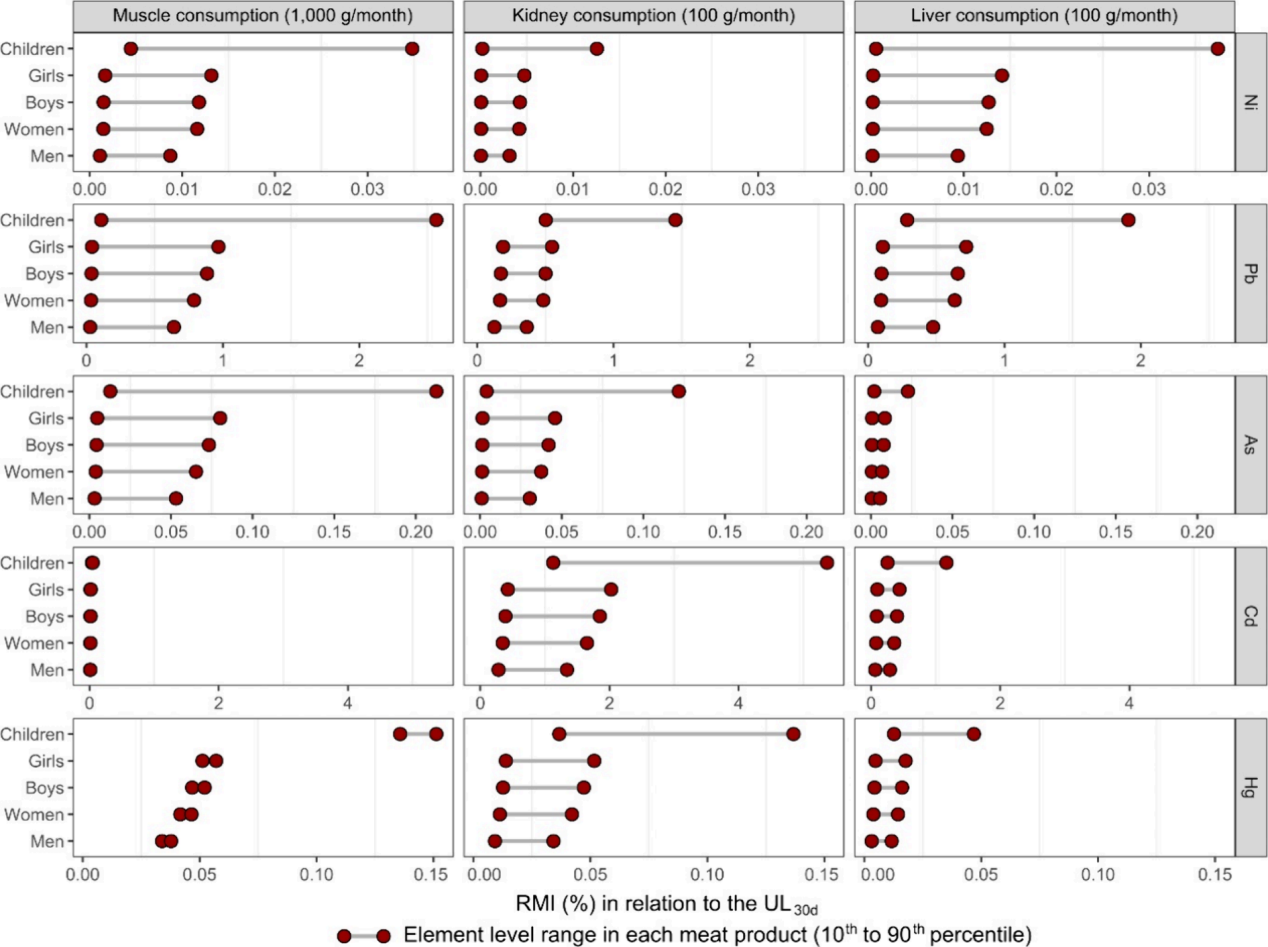
Estimate of relative monthly intake (RMI) in relation to the tolerable upper levels adjusted to 30 days (UL_30d_) for human categories by consumption of the muscle, kidney, and liver of cattle obtained in markets located in the southern region of the Amazon. The following ages and body weights were assumed for human categories: 6 years and 20 kg for children; 16 years and 53 kg for girls; 16 years and 58 kg for boys; 30 years and 65 kg for women; and 30 years and 80 kg for men. Element concentrations in the meat products in this figure were the range between the 10th and 90th percentile of the descriptive results for the muscle, kidney, and liver (Tables S8–S10).

## 4 Discussion

The concentrations of micronutrients or toxic elements in the soil obtained by pseudototal analysis provide indications of the soil health status and are important for environmental assessment.^34^ Thus, very high levels may indicate contamination, while low levels usually reflect naturally low levels in the rock. In the present study, the high levels of Fe in the soil are related to the genesis of Latosols and Argisols in the tropical region,^48^ and they do not therefore represent contamination. Low levels of some micronutrients, e.g., Zn and Cu, indicate possible deficiencies of these elements for the growth and establishment of pastures.^41^ Furthermore, the continued use of pastures for grazing animals, associated with low rates of nutrient replacement, leads to soil exhaustion and to low productivity and degradation of pasture.^33^

The high levels of Se, Mo, and Hg levels in some of the soils probably reflect variations in the pedogenetic processes in this region, although the levels are usual for world soils. No fertilizer inputs of Se or Mo were reported on any of the farms, which suggest that these elements occur naturally. For Brazilian soils, levels of up to 5 mg/kg of Se and 30 mg/kg of Mo (i.e., much higher than observed in this study) are considered safe.^49^ There is particular concern regarding Hg levels due to the risk of exposure from artisanal mining and fires in this region.^50,51^ For Brazilian soils, Hg levels below 0.5 mg/kg are considered safe.^49^ However, Hg levels up to 0.96 mg/kg have been reported for uncontaminated soils in the Amazon.^52^ Therefore, Hg levels reaching 0.24 mg/kg on these farms appear natural, and they are similar to natural levels reported for several Amazon sites.^53^

The available levels of micronutrients for plants in relation to pseudototal levels generally represent small fractions, which vary depending on several factors, e.g., soil mineralogy, pH, and soil organic matter.^54^ Thus, even elements typically present at high levels in tropical soils (as determined by pseudototal analysis) may be present at deficient levels for plant nutrition.^55^ In this context, it is important to highlight that soil should be appropriately managed to provide an adequate supply of nutrients for the establishment of these pastures, mainly Zn and Cu, which are typically deficient in these soils.

Overall, despite the predominance of weak correlations of elements between soil and grass (Figure S2), the elements in the grass were proportional to those in the soil. In summary, adequate levels of Mn or deficient levels of Zn, Cu, Se, Co, and Mo in grass can be explained by the levels of these elements in the soil. The element content of the soil should be taken into consideration when planning nutritional supplementation for the herd. Thus, excessive supplementation can be reduced when the pasture already meets the animal’s requirements, or the supply can be improved where deficiencies are severe. In relation to Fe levels, although this element occurs at high levels in the soil, zebu beef cattle also have high Fe requirements; therefore, under these conditions, Fe supplementation should be continued.

A wide variety of nutritional supplements are provided to cattle in the Amazon. Nonetheless, sampling carried out on farms enabled us to observe some aspects related to mineral supplementation. In conditions where pasture is severely deficient in micronutrients such as Cu and Zn, some supplements do not meet the animal’s requirements, while others provide much higher amounts, approaching or even exceeding the maximum limits tolerated by the animals. Furthermore, the soils and pastures are also much more variable throughout the Amazon region than in the study region, highlighting the need to develop cattle nutrition strategies at the herd level. Thus, the generic animal nutrition recommendations typically assumed for the Amazon region are often inadequate for real grazing conditions.

Assessment of cattle nutritional status by blood plasma analysis revealed low Mo and Se levels in herds grazing in areas where these elements were poor in the soil and grass. The low level of Mo in blood plasma does not appear to be problematical, as the concentrations of this element are not associated with deficiencies in practical conditions.^19,56^ Furthermore, low Mo levels in the diet can favor the absorption of Cu, which is present at extremely low levels in these pastures. However, ensuring that Cu supplementation does not exceed the tolerated limit of 40 mg/kg DM of the diet is particularly important in these conditions to prevent the risk of Cu poisoning where Mo is present at very low levels. For example, Cu poisoning has been reported in pasture-fed Nellore cattle, leading to the death of several animals that consumed high amounts of supplements with Cu levels of 50 mg/kg.^57^

The low Se levels in the blood plasma of some cattle indicate that the nutritional supplements did not provide an adequate supply of this element in the diet. This probably occurred because of the low bioavailability of mineral sources of Se. The bioavailability of inorganic sources of Se, such as selenate (SeO_4_^–2^) and selenite (SeO_3_^–2^), is low in cattle, and organic Se sources are therefore more suitable.^23,58^ This is consistent with the findings of this study, as the cattle grazing in the area where the Se content in grass was highest were the only ones with adequate Se status. The importance of soil for supplying nutrients to the herd can also be highlighted (Figure S3B).

The levels of the toxic elements Pb, Cd, As, and Hg in the blood plasma were normal. The high correlation between Hg in soil and in grass (Figure S3A) resulted in only very low levels of Hg in the grass; consequently, it did not affect blood Hg levels. However, high levels of these elements in the blood are generally observed only in cases of chronic or acute poisoning.^59^ Analysis of accumulating organs, such as the kidney and liver, is useful for assessing the level of environmental exposure of animals to these elements. Although it was not possible to obtain kidney and liver samples from the animals under study, we assumed that samples obtained in markets in the region were representative of the risks of environmental contamination to which these animals are exposed.

Therefore, we observed that the Pb, As, Cd, and Hg levels in the kidney and liver samples obtained from the markets located in the southern region of the Amazon (Figure 7) were generally lower than the levels reported in several countries^60–67^ including Brazil.^68^ This finding indicates that cattle raised in this region are exposed to low levels of these toxic elements and can be attributed to the diet, which generally includes pasture with very low levels of these elements. The cattle are usually fed pasture and given small amounts of nutritional supplements until they complete the growth phase and begin the carcass finishing phase, i.e., at around 24 to 30 months of age. They are then fed more concentrated diets for a short time (about 3 months), sometimes while still on pasture or in feedlot, until it reaches slaughter weight.

The present study findings indicate the importance of geochemical studies of soils and trace element concentrations in grasses as well as monitoring byproducts and feed offered to livestock. This type of information can support more efficient management of animal nutrition, also taking into account regional production arrangements for raising cattle or other animal species, such as sheep, poultry, and pigs.

Studies evaluating the animal nutritional status often relate the results obtained to environmental factors. For example, hair samples are often analyzed to assess exposure to trace elements in various species of animals, as hair responds to changes in environmental conditions such as soil type, vegetation, and water.^69–71^ Blood and liver are also valuable for assessing the nutritional status of cattle and their relationship with the environment.^9^ Thus, considering environmental information in a farm management plan can contribute to more efficient production and provide safe food.

When considering the concentrations of micronutrients or toxic elements analyzed in meat products for human consumption, the RDI_30d_ or UL_30d_ provides relevant information. Thus, the choice of a food can be favored by its potential contribution to a healthier diet. Consumption of offal has been suggested as a good option for providing nutrients and reducing environmental impacts.^72,73^ However, consumers may be reluctant to consume this type of food.^74^

Regarding the micronutrient contents of the muscle, kidney, and liver samples, these products can be considered rich sources of various elements for human nutrition. The muscle generally has lower levels of elements than the offal (kidney and liver); however, high levels of Cr, Zn, and Fe were detected in the muscle. Additionally, the low levels of toxic elements in these meat products help to enhance their nutritional value. The concentrations of elements in the offal, which are known to accumulate toxic elements, did not reach levels that would represent a significant threat to human health.

No previous studies have reported human or animal health problems related to trace elements and pasture conditions in the Amazon, apart from micronutrient deficiencies (which are of global concern). However, some attention may be required regarding Pb and Cd in the muscle, kidney, and liver of cattle, in which the highest UL_30d_ values were reached. Although it was not possible to determine the age of the animals from which meat product samples were obtained, these high levels can probably be attributed to samples being from older animals. In this region of the Amazon, it is estimated that more than 25% of animals are slaughtered at ages over 36 months old.^75^ Thus, strategies that reduce the average age at which animals are slaughtered could reduce the time of exposure to bioaccumulation and the levels of these elements in the meat products. It is also important to ensure the supply of nutritional supplements with low levels of toxic elements in livestock feed. Furthermore, monitoring soil and pasture could help to identify areas where higher levels of these elements may occur and thus identify areas with a greater risk of environmental exposure. For example, soils of igneous or metamorphic lithology in southern Amazonia have been reported to contain high levels of Cd, reaching up to 2 mg/kg.^76^ Thus, in these areas, producers and authorities should implement measures for monitoring and preventing environmental exposure to Cd.

The findings of this study contribute to understanding the importance of rational use of soils, pastures, and mineral supplementation in pasture-based animal production systems. Natural variations in micronutrient levels in the environment were also found to have strong effects on the balance of dietary formulations for cattle raised under these conditions. The development of sustainable strategies for pasture systems must therefore consider, among the many social, economic, and environmental factors involved, the interactions between micronutrients and toxic elements across the environmental–animal–human compartments.

In conclusion, we highlight the importance of applying the One Health approach in considering the different components of pasture-based systems to produce foods with low levels of toxic elements and useful levels of micronutrients and to safeguard environmental, animal, and human health. The study findings show that careful management of trace element concentrations throughout the production system is essential for supplying safe food to consumers. This management approach includes considering environmental protection with rational use of land and natural resources, such as those used to produce animal feed. In addition, attention to animal health and welfare is required to ensure an adequate supply of nutrients in animal diet.

## Supplementary Material



## Notes

The Supporting Information provided in tabular form includes general farm information, description of nutritional supplements of cattle, LODs of the chemical analysis, estimated element intake by cattle, descriptive statistics of element levels in meat products, and dietary reference values for some human groups; other information is provided in graphic format, including a boxplot of available levels of Fe, Mn, Zn, and Cu in soil, a correlation matrix of element levels between soil and grass, and a regression plot for Hg and Se in soil and grass (PDF)

## Abbreviations

AU: animal unit of 450 kg body weight
LOD: limit of detection
DM: dry matter
ICP-MS: inductively coupled plasma mass spectrometry
RDI_30d_: reference daily intake adjusted to 30 days
RMI: relative monthly intake
UL_30d_: tolerable upper level adjusted to 30 days
