# Supplementary material for: Micronutrients and Toxic Elements in Soil, Grass, and Nutritional Supplements and in Blood and Meat Products from Beef Cattle Raised in the Southern Amazon, Brazil
Source: J Agric Food Chem. 2025 Jun 10;73(25):15500–13. doi: 10.1021/acs.jafc.4c12513 (PMC12203612; doi:10.1021/acs.jafc.4c12513)
Supplement: Supplementary file 1 [file jf4c12513_si_001.pdf]

Supporting Information

Micronutrients and toxic elements in soil, grass and nutritional supplements and in blood and meat products from beef cattle raised in the southern Amazon, Brazil

Fernando Luiz Silva[1]*, Marcus Henrique Martins e Silva[1], Ernandes Sobreira Oliveira Júnior[2], Áurea Regina Alves Ignácio[2], Marta López-Alonso[3], Marta Miranda[4], Veronica Piñeiro[5], Maria Aparecida Pereira Pierangeli[6]

*Email: fernando.silva@ifmt.edu.br

1. Department of Education, Federal Institute of Mato Grosso, Alta Floresta 78580-000, MT, Brazil;

2. Center for Research on Limnology, Biodiversity, and Ethnoecology, Graduate Program of Environmental Science, University of Mato Grosso State, Cáceres, MT, 78200-000, Brazil;

3. Department of Animal Pathology, Faculty of Veterinary, Campus Terra, University of Santiago de Compostela, 27002 Lugo, Spain;

4. Department of Anatomy, Animal Production and Veterinary Clinical Sciences, Faculty of Veterinary, Campus Terra, University of Santiago de Compostela, 27002 Lugo, Spain;

5. Instrumental Analysis Unit, Network of Infrastructures to Support Research and Technological Development (RIAIDT), Campus Terra, University of Santiago de Compostela, 27002 Lugo, Spain;

6. Department of Animal Science, Graduate Program of Environmental Science, University of Mato Grosso State, Pontes e Lacerda, MT, 78250-000, Brazil.

Table S1. General information on farms and Nellore female herds of this study.

Farm/herd	Soil class[A]	Pasture grass	Age of cattle (months)
I	Argisol and Latosol	*Urochloa brizantha*	42 ± 6
II	Argisol and Latosol	*U. brizantha and Megathyrsus maximus*	60 ± 12
III	Argisol and Latosol	*U. brizantha and M. maximus*	18 ± 2
IV	Latosol	*U. brizantha*	21 ± 3
V	Argisol and Latosol	*U. brizantha*	-
VI-a	Argisol	*U. brizantha*	18 ± 1
VI-b	Argisol	*U. brizantha*	17 ± 1
VII	Argisol and Latosol	*U. brizantha*	-
VIII	Argisol and Latosol	*M. maximum*	-

[A] Brazilian system of soil classification [1].

Table S2. Description of the recommended daily consumption of each nutritional supplement and calculated amount of grass daily consumption considering pasture-fed zebu beef cattle of body weight equivalent to 450 kg.

Nutritional supplement	Recommended consumption of the supplement	DM	Estimated supplement consumption[A]	Estimated grass consumption[B]
	(g/AU NM per day)	(%)	(g/AU DM per day)	
Protein/energy (id 1)	2,700	90.7	2,448.0	7,052.0
Protein/energy (id 2)	1,000	92.3	923.3	8,576.7
Mineral block (id 3)	450	82.2	370.0	9,130.0
Protein/energy (id 4)	500	90.3	451.7	9,048.3
Protein/energy (id 5)	1,000	91.3	913.3	8,586.7
Protein/energy (id 6)	1,000	91.7	916.7	8,583.3
Protein/energy (id 7)	500	92.0	460.0	9,040.0
Protein/energy (id 8)	500	90.7	453.3	9,046.7
Mineral block (id 9)	100	86.7	86.7	9,413.3
Protein/energy (id 10)	2,700	90.0	2,430.0	7,070.0
Protein/energy (id 11)	1,350	91.3	1,233.0	8,267.0
Corn silage (id 12)	15,000	32.3	4,845.0	4,655.0
Mineral powder (id 13)	100	97.2	97.2	9,402.8
Mineral powder (id 14)	135	96.2	129.8	9,370.2
Mineral powder (id 15)	100	96.5	96.5	9,403.5
Mineral powder (id 16)	100	95.8	95.8	9,404.2
Mineral powder (id 17)	100	96.5	96.5	9,403.5
Mineral powder (id 18)	135	95.3	128.7	9,371.3
Mineral powder (id 19)	135	95.5	128.9	9,371.1
Mineral powder (id 20)	100	96.0	96.0	9,404.0
Mineral premix[C] (id 21)	-	98.4	-	-
Mineral premix[C] (id 22)	-	97.6	-	-

Abbreviations: AU = animal unit of 450 kg body weight; DM = dry matter; NM = natural matter. [A] The assumed daily amount consumed of each nutritional supplement was equivalent to the recommendation presented on the label of each sampled product, calculated on a DM basis; [B] The assumed daily amount of grass consumed was the difference between the assumed total daily consumption (9.5 kg) and the daily consumption of each nutritional supplement. [C] Mineral premix is not consumed directly by cattle.

Table S3. Limits of detection (LOD) , in µg/L, obtained in each batch of analyses using the inductively coupled plasma mass spectrometry (ICP-MS) technique.

Element	Soil	Grass and nutritional supplements	Blood plasma	Meat products
Fe	<0.055	<0.059	2.315	8.061
Mn	<0.019	0.045	0.049	0.129
Zn	<0.178	<0.275	<0.189	1.941
Cu	<0.009	0.061	<0.008	<0.097
Se	<0.003	<0.041	0.034	0.383
Co	<0.009	0.004	0.017	<0.005
Mo	<0.007	0.007	0.020	0.014
Ni	<0.008	<0.004	0.038	<0.015
Cr	<0.018	0.008	0.101	0.195
Cd	<0.009	0.008	<0.006	<0.007
Pb	<0.005	0.058	0.071	0.028
As	<0.011	<0.012	<0.036	<0.030
Hg	0.039	0.038	<0.012	<0.022

Table S4. Dietary reference values for different target groups, adjusted to 30 days.

Element	Reference	Target group^{A}				
		Children	Girls	Boys	Women	Men
		RDI_{30d} (mg)				
Fe	Institute of Medicine [2]	300.0	450.0	330.0	540.0	240.0
Mn	Institute of Medicine [2]	45.0	48.0	66.0	54.0	69.0
Zn	Institute of Medicine [2]	150.0	270.0	330.0	240.0	330.0
Cu	Institute of Medicine [2]	13.2	26.7	26.7	27.0	27.0
Se	Institute of Medicine [2]	0.90	1.65	1.65	1.65	1.65
Co^{B}	-	-	-	-	-	-
Mo	Institute of Medicine [2]	0.66	1.29	1.29	1.35	1.35
Cr	Institute of Medicine [2]	0.45	0.72	1.05	0.75	1.05
		UL_{30d} (mg)				
Ni^{C}	Schrenk et al. [3]	7.80	20.67	23.01	23.40	31.20
Pb^{D}	FAO/WHO [4]	0.18	0.48	0.52	0.59	0.72
As	FAO/WHO [5]	1.80	4.77	5.22	5.85	7.20
Cd	Alexander et al. [6]	0.21	0.57	0.62	0.70	0.86
Hg	FAO/WHO [5]	0.34	0.91	0.99	1.11	1.37

Abbreviations: RDI_{30d} = Reference daily intake, adjusted to 30 days; UL_{30d} = Tolerable upper level, adjusted to 30 days. ^{A} The following different groups were considered: children, 6 years old, 20 kg; girls, 16 years old, 53 kg; boys, 16 years old, 58 kg, women, 30 years old, 65 kg; and men, 30 years old, 80 kg; ^{B} There is no reference daily intake for Co. This element is required for humans only as vitamin B12; ^{C} Although Ni is required as a nutrient, there is no recommended daily intake for this element; ^{D} To calculate the Pb UL_{30d}, the value was assumed to be 0.3 µg/kg body weight per day, considered an insignificant risk for children, according to FAO/WHO (2011).

Table S5. Estimate of Fe, Mn, Zn and Cu intake by zebu beef cattle (450 kg body weight) consuming 9.5 kg of dry matter (DM) per day, considering the combined consumption of nutritional supplement and grass, on farms located in the southern region of the Amazon.

Nutritional supplement	Supplement consumption	Grass consumption	Estimated intake of elements [A]							
	kg DM per day		Fe (mg/kg)		Mn (mg/kg)		Zn (mg/kg)		Cu (mg/kg)	
Protein/energy (id 1)	2.448	7.052	106.02	333.74	94.08	394.57	93.48	105.77	57.90	61.47
Protein/energy (id 2)	0.923	8.577	120.07	397.03	75.36	440.81	106.44	121.39	22.30	26.64
Mineral block (id 3)	0.370	9.130	78.57	373.38	63.80	452.83	38.86	54.78	11.60	16.22
Protein/energy (id 4)	0.452	9.048	145.31	437.49	74.86	460.41	46.73	62.50	16.79	21.37
Protein/energy (id 5)	0.913	8.587	153.09	430.37	69.79	435.67	88.80	103.77	17.79	22.14
Protein/energy (id 6)	0.917	8.583	196.70	473.87	74.03	439.76	95.09	110.05	26.88	31.22
Protein/energy (id 7)	0.460	9.040	91.79	383.71	71.73	456.93	33.02	48.78	11.09	15.66
Protein/energy (id 8)	0.453	9.047	93.67	385.80	74.57	460.05	33.11	48.88	9.65	14.23
Mineral block (id 9)	0.087	9.413	74.88	378.84	81.35	482.45	38.94	55.35	12.75	17.51
Protein/energy (id 10)	2.430	4.655	396.57	624.87	131.74	432.99	182.70	195.03	58.51	62.08
Protein/energy (id 11)	1.233	8.267	64.07	331.02	61.87	414.13	21.10	35.51	4.91	9.09
Corn silage (id 12)	4.845	4.655	359.67	509.98	51.36	249.71	47.96	56.08	14.73	17.08
Mineral powder (id 13)	0.097	9.403	121.54	425.17	83.51	484.17	64.87	81.26	16.06	20.82
Mineral powder (id 14)	0.130	9.370	113.62	416.19	75.87	475.13	52.94	69.28	11.59	16.33
Mineral powder (id 15)	0.097	9.404	101.50	405.15	73.19	473.87	48.22	64.61	13.71	18.47
Mineral powder (id 16)	0.096	9.404	115.68	419.36	78.96	479.67	58.79	75.18	15.80	20.56
Mineral powder (id 17)	0.097	9.404	101.29	404.95	88.06	488.74	94.97	111.36	20.84	25.60
Mineral powder (id 18)	0.129	9.371	181.45	484.06	77.00	476.31	51.06	67.39	17.05	21.79
Mineral powder (id 19)	0.129	9.371	169.64	472.24	81.47	480.77	56.37	72.71	22.13	26.87
Mineral powder (id 20)	0.096	9.404	80.90	384.57	79.47	480.18	53.80	70.19	20.29	25.05
Requirement for beef cattle [7]			40.0		20.0		30.0		10.0	
Requirement for beef zebu beef cattle [8] [B]			194.61		21.43		46.65		7.13	
Maximum tolerable [7]			500.0		1,000.0		500.0		40.0	

[A] This estimate considers a range between the minimum and maximum levels of each element in the grass, in mg/kg DM, as follows: Fe 43.07-349.83; Mn 62.50-467.30; Zn 11.43-27.99; and Cu 2.77-7.57; [B] The animal group considered for calculation of the intake was pasture-raised, non-castrated male zebu beef cattle of body weight 450 kg and an average daily weight gain of 0.8 kg.

Table S6. Estimated Se, Co, Mo, Cr, and Ni intake by zebu beef cattle (450 kg body weight) consuming 9.5 kg of dry matter (DM) per day, considering the combined consumption of nutritional supplement and grass, on farms located in the southern region of the Amazon.

Nutritional supplement	Supplement consumption	Grass consumption	Estimated range of intake of elements[A]									
	kg DM per day		Se (mg/kg)		Co (mg/kg)		Mo (mg/kg)		Cr (mg/kg)		Ni (mg/kg)	
Protein/energy (id 1)	2.448	7.052	1.00	1.11	2.21	2.23	0.23	1.21	0.59	1.63	0.37	0.84
Protein/energy (id 2)	0.923	8.577	0.67	0.80	2.61	2.64	0.12	1.31	0.69	1.96	0.29	0.87
Mineral block (id 3)	0.370	9.130	0.18	0.32	0.58	0.61	0.06	1.33	0.51	1.86	0.21	0.83
Protein/energy (id 4)	0.452	9.048	0.20	0.34	0.58	0.60	0.07	1.33	0.61	1.95	0.39	1.00
Protein/energy (id 5)	0.913	8.587	0.29	0.42	1.17	1.19	0.10	1.30	0.59	1.86	0.41	0.99
Protein/energy (id 6)	0.917	8.583	0.41	0.54	1.43	1.45	0.09	1.29	0.71	1.98	0.49	1.06
Protein/energy (id 7)	0.460	9.040	0.10	0.24	0.81	0.83	0.05	1.31	0.42	1.76	0.27	0.88
Protein/energy (id 8)	0.453	9.047	0.19	0.32	0.12	0.15	0.08	1.34	0.58	1.92	0.27	0.88
Mineral block (id 9)	0.087	9.413	0.21	0.35	0.12	0.15	0.05	1.36	1.03	2.42	0.23	0.86
Protein/energy (id 10)	2.430	4.655	1.30	1.41	4.57	4.59	0.17	1.16	1.15	2.20	0.95	1.42
Protein/energy (id 11)	1.233	8.267	0.12	0.25	0.32	0.35	0.15	1.31	0.41	1.63	0.20	0.76
Corn silage (id 12)	4.845	4.655	0.52	0.59	1.24	1.26	0.10	0.75	0.72	1.41	0.26	0.58
Mineral powder (id 13)	0.097	9.403	0.18	0.33	0.99	1.01	0.05	1.36	0.80	2.19	0.38	1.01
Mineral powder (id 14)	0.130	9.370	0.19	0.33	1.11	1.14	0.05	1.36	0.60	1.99	0.25	0.88
Mineral powder (id 15)	0.097	9.404	0.29	0.43	0.63	0.66	0.05	1.36	0.48	1.87	0.29	0.92
Mineral powder (id 16)	0.096	9.404	0.21	0.36	0.79	0.81	0.05	1.37	0.52	1.91	0.35	0.98
Mineral powder (id 17)	0.097	9.404	0.45	0.59	1.19	1.21	0.05	1.36	0.64	2.04	0.34	0.97
Mineral powder (id 18)	0.129	9.371	0.20	0.35	0.88	0.90	0.06	1.37	1.23	2.61	0.46	1.09
Mineral powder (id 19)	0.129	9.371	0.20	0.34	1.02	1.04	0.06	1.37	1.17	2.56	0.36	0.99
Mineral powder (id 20)	0.096	9.404	0.27	0.41	1.51	1.53	0.05	1.37	0.63	2.02	0.25	0.89
Requirement for beef cattle [7]			0.1		0.15		-		-		-	
Requirement for beef zebu beef cattle [8] [B]			0.48		0.74		0.32		2.26		-	
Maximum tolerable [7]			5.0		25.0		5.0		1,000.0		5.0	

[A] This estimate considers a range between the minimum and maximum levels of each element in the grass, in DM as follows: Se 0.79-145.94 µg/kg; Co 4.19-29.51 µg/kg; Mo 35.31-1,362.71 µg/kg; Cr 0.38-1.79 mg/kg; and Ni 0.14-0.78 µg/kg; [B] The animal group considered for calculation of the intake was pasture-raised, non-castrated male zebu beef cattle of body weight 450 kg and an average daily weight gain of 0.8 kg.

Table S7. Estimated intake of Pb, As, Cd and Hg by zebu beef cattle (450 kg body weight) consuming 9.5 kg of dry matter (DM) per day, considering the combined consumption of nutritional supplement and grass, on farms located in the southern region of the Amazon.

Nutritional supplement	Supplement consumption	Grass consumption	Estimated range of intake elements[A]							
	kg DM per day		Pb (µg/kg)		As (µg/kg)		Cd (µg/kg)		Hg (µg/kg)	
Protein/energy (id 1)	2.448	7.052	79.19	678.24	46.27	51.26	3.55	17.99	8.71	10.69
Protein/energy (id 2)	0.923	8.577	97.21	825.78	56.19	62.27	16.31	33.87	1.53	3.94
Mineral block (id 3)	0.370	9.130	89.00	864.56	25.12	31.59	8.00	26.69	1.06	3.63
Protein/energy (id 4)	0.452	9.048	95.26	863.89	69.80	76.21	32.30	50.81	1.32	3.86
Protein/energy (id 5)	0.913	8.587	103.32	832.73	66.63	72.71	36.12	53.69	1.02	3.43
Protein/energy (id 6)	0.917	8.583	110.78	839.91	92.48	98.56	43.45	61.01	2.45	4.86
Protein/energy (id 7)	0.460	9.040	72.77	840.69	22.11	28.52	2.09	20.59	1.12	3.66
Protein/energy (id 8)	0.453	9.047	76.33	844.82	25.74	32.14	5.37	23.89	1.21	3.75
Mineral block (id 9)	0.087	9.413	76.67	876.31	63.32	69.99	8.16	27.42	1.09	3.73
Protein/energy (id 10)	2.430	4.655	102.52	703.10	290.66	295.67	10.18	24.65	6.92	8.23
Protein/energy (id 11)	1.233	8.267	68.54	770.80	12.81	18.67	1.46	18.38	2.06	4.38
Corn silage (id 12)	4.845	4.655	123.98	519.41	38.50	41.80	2.84	12.36	1.82	3.13
Mineral powder (id 13)	0.097	9.403	91.81	890.56	56.81	63.47	32.19	51.44	1.09	3.73
Mineral powder (id 14)	0.130	9.370	91.81	887.78	44.45	51.09	10.63	29.81	1.08	3.71
Mineral powder (id 15)	0.097	9.404	114.15	912.95	26.42	33.09	4.99	24.23	2.22	4.86
Mineral powder (id 16)	0.096	9.404	108.84	907.70	40.59	47.25	20.50	39.74	1.48	4.12
Mineral powder (id 17)	0.097	9.404	105.56	904.36	54.20	60.86	18.56	37.80	1.10	3.74
Mineral powder (id 18)	0.129	9.371	138.10	934.17	63.78	70.41	42.70	61.88	1.12	3.75
Mineral powder (id 19)	0.129	9.371	130.26	926.30	48.75	55.39	48.65	67.83	1.14	3.77
Mineral powder (id 20)	0.096	9.404	81.73	880.57	32.57	39.23	10.44	29.69	1.09	3.73
Requirement for beef cattle [7]			-		-		-		-	
Requirement for beef zebu beef cattle [8] [B]			-		-		-		-	
Maximum tolerable [7]			30,000.0		50,000.0		500.0		2,000.0	

[A] This estimate considers a range between the minimum and maximum levels of each element in the grass, in µg/kg DM, as follows: Pb 67.77-874.77; As 0.39-14.88; Cd 0.33-19.78; and Hg 1.07-3.74;
[B] The animal group considered for calculation of the intake was pasture, raised non-castrated male zebu beef cattle, of body weight 450 kg and with an average daily weight gain of 0.8 kg.

Table S8. Descriptive statistics of micronutrients and toxic elements in the muscle of cattle obtained in markets located in the southern region of the Amazon.

Element	Unit	Average	SD	Percentile						
				Minimum	10th	25th	50th	75th	90th	Maximum
Fe	mg/kg	19.9416	4.3672	12.6618	14.7759	16.8507	19.4533	21.7123	26.2637	29.2572
Mn		0.0653	0.0105	0.0487	0.0506	0.0581	0.0658	0.0722	0.0784	0.0885
Zn		260.7393	54.9638	169.4757	208.7558	228.5975	245.5108	287.4511	316.4971	440.7871
Cu		0.4834	0.0688	0.3500	0.4001	0.4486	0.4771	0.5014	0.5969	0.6515
Se		0.0664	0.0304	0.0235	0.0326	0.0513	0.0590	0.0745	0.1140	0.1475
Co		0.0023	0.0011	0.0007	0.0010	0.0016	0.0022	0.0028	0.0038	0.0057
Mo		0.0078	0.0100	0.0010	0.0031	0.0035	0.0049	0.0070	0.0118	0.0550
Cr	µg/kg	68.2329	69.6600	3.7802	10.4112	13.4582	34.7333	114.4737	178.9322	219.4583
Ni		2.0403	3.6860	0.0115	0.3466	0.5561	1.4634	2.2281	2.7162	21.4913
Pb		1.6921	2.7319	0.1320	0.1948	0.2032	0.5076	1.8817	4.6168	13.5838
As		1.4928	1.7450	0.0528	0.2313	0.4346	0.7680	1.6685	3.8253	6.7511
Cd		0.1148	0.1334	0.0741	0.0796	0.0805	0.0844	0.0879	0.1058	0.8168
Hg		0.4831	0.0680	0.1609	0.4654	0.4693	0.4887	0.5040	0.5183	0.6263

Abbreviation: SD = Standard Deviation.

Table S9. Descriptive statistics of micronutrients and toxic elements in the kidney of cattle obtained in markets located in the southern region of the Amazon.

Element	Unit	Average	SD	Percentile						
				Minimum	10th	25th	50th	75th	90th	Maximum
Fe	mg/kg	65.3461	14.8677	39.6513	46.4977	55.0025	65.2606	73.6838	79.1960	106.7746
Mn		1.0761	0.1677	0.7766	0.9201	0.9714	1.0535	1.1495	1.3070	1.5383
Zn		137.2765	16.2593	112.7711	119.4794	124.9385	133.2810	149.1110	160.3916	172.4651
Cu		3.3757	0.6678	2.4315	2.6662	2.8966	3.2950	3.6972	4.2019	4.9614
Se		1.0172	0.1617	0.7204	0.8239	0.9244	1.0158	1.1033	1.2330	1.3817
Co		0.0555	0.0209	0.0259	0.0305	0.0345	0.0539	0.0702	0.0762	0.1199
Mo		0.3000	0.0952	0.0869	0.1326	0.2550	0.3397	0.3637	0.3823	0.4124
Cr	μg/kg	8.8098	8.5079	0.6635	1.2144	2.7131	7.0533	9.5975	21.5163	34.7903
Ni		3.5354	5.9216	0.0410	0.1602	0.4356	1.4142	2.8480	9.8080	28.5938
Pb		17.7183	7.0823	6.3192	9.0445	12.4323	17.7537	20.8419	26.1644	32.7770
As		7.0904	8.0021	0.3318	0.7186	1.7599	3.1355	10.0285	21.9116	24.5495
Cd		58.7686	46.6571	12.0101	24.1812	26.8442	41.0676	71.4460	115.0263	223.7759
Hg		2.6038	1.4462	0.5082	1.2579	1.7290	2.3516	2.9203	4.6918	6.8953

Abbreviation: SD = Standard Deviation.

Table S10. Descriptive statistics of micronutrients and toxic elements in the beef liver samples obtained from markets located in the southern region of the Amazon.

Element	Unit	Average	SD	Percentile						
				Minimum	10th	25th	50th	75th	90th	Maximum
Fe	mg/kg	84.3674	29.3680	40.1246	53.5715	63.1581	79.7173	95.6644	118.0720	182.4773
Mn		2.7925	0.4135	2.0559	2.2529	2.5529	2.7435	3.0749	3.4067	3.6459
Zn		278.3077	51.6385	192.9775	216.6842	255.6737	271.2259	296.6441	332.6995	444.5566
Cu		132.3123	64.7942	24.8131	63.0866	91.9875	124.0057	157.6703	213.5300	330.7441
Se		0.2987	0.1257	0.1600	0.1890	0.2253	0.2683	0.2999	0.4973	0.6539
Co		0.0787	0.0463	0.0167	0.0283	0.0482	0.0725	0.1028	0.1201	0.1973
Mo		0.8989	0.2536	0.1283	0.6534	0.7641	0.9498	1.0288	1.1360	1.3320
Cr	µg/kg	72.4783	83.5253	3.2755	6.0372	12.1589	51.4683	92.7492	141.6726	355.1919
Ni		9.7693	13.0628	0.2142	0.4125	1.8288	5.3405	10.2982	29.1859	53.2683
Pb		16.7695	11.9718	1.5714	5.1723	9.0215	12.5841	22.1424	34.3754	50.1475
As		1.7098	2.0741	0.2137	0.3720	0.5882	0.7381	2.1250	4.0968	9.3062
Cd		15.2850	12.0105	3.4948	5.4518	8.1052	12.6007	18.4798	25.0039	56.0180
Hg		0.8284	0.9192	0.0368	0.4362	0.5411	0.5599	0.5837	1.6077	4.0082

Abbreviation: SD = Standard Deviation.

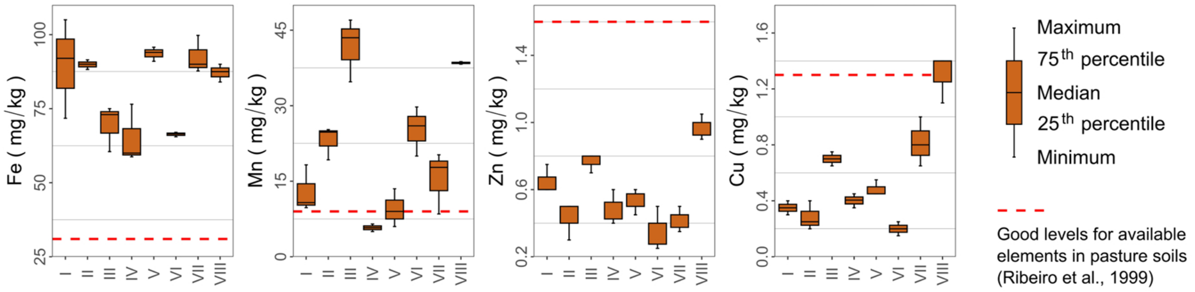

Figure S1. Descriptive statistics of available elements (extracted with Mehlich-1 solution) in the soil on farms I-VIII, located in the southern region of the Amazon.

Trace elements in the grass (columns) × Trace elements in the soil (rows)

	Fe	Mn	Zn	Cu	Se	Co	Mo	Cr	Ni	Pb	As	Cd	Hg
Fe	** 0.61	-0.24	** -0.54	-0.22	-0.04	-0.17	-0.22	-0.39	-0.3	* -0.44	-0.03	* -0.46	* -0.43
Mn	-0.13	0.14	0.07	-0.16	0.32	0.11	-0.4	-0.17	-0.27	-0.19	** -0.54	0.04	* 0.44
Zn	-0.39	0	0.15	-0.31	0.07	-0.08	-0.39	-0.14	-0.31	0.05	** -0.55	0.05	** 0.62
Cu	* 0.51	0.01	-0.15	0.07	0.26	0.23	-0.17	-0.2	-0.12	** -0.57	-0.25	-0.25	-0.2
Se	0.05	*** 0.77	0.2	* 0.48	*** 0.91	** 0.57	-0.16	0.17	** 0.52	-0.04	0.11	-0.09	* 0.42
Co	-0.11	0.09	0.2	-0.08	0.06	0.11	-0.33	-0.17	-0.27	-0.04	* -0.42	0.34	-0.01
Mo	0.24	-0.12	-0.41	-0.11	-0.04	-0.26	* 0.49	-0.24	-0.06	-0.11	0.13	-0.35	* -0.43
Cr	0.41	-0.24	-0.2	-0.02	-0.03	0.02	-0.15	-0.25	-0.29	** -0.52	-0.28	-0.27	-0.21
Ni	* 0.47	-0.14	-0.21	-0.08	0.05	0.09	-0.07	-0.2	-0.2	** -0.59	-0.33	-0.31	-0.2
Pb	-0.34	-0.17	-0.27	-0.34	-0.14	** -0.54	* -0.45	* -0.49	* -0.5	0.2	-0.31	-0.08	0.06
As	0.1	0.02	-0.27	0.03	0.05	-0.1	0.32	-0.03	0.1	-0.15	0.1	-0.33	-0.3
Cd	0.13	* -0.43	* -0.5	-0.39	* -0.46	** -0.52	0.09	-0.29	-0.39	-0.05	0.09	-0.19	* -0.48
Hg	-0.21	* 0.45	0	-0.06	** 0.61	0.28	-0.24	-0.07	-0.03	-0.3	* -0.43	-0.23	*** 0.94

Pearson's rank correlations: * $p \leq 0.05$; ** $p \leq 0.01$; *** $p \leq 0.001$

Colour scale: -1, -0.5, 0, 0.5, 1

Figure S2. Pearson's rank correlations between elements in the soil and grass in farms in the southern region of the Amazon.

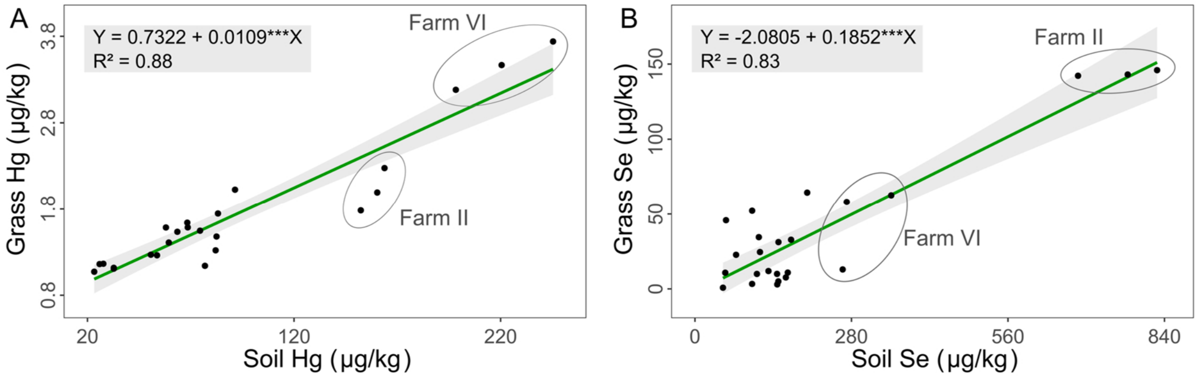

***: significant at $p \leq 0.001$ by F test

Figure S3. Regression between Hg in soil and grass (A), and Se in soil and grass (B) on farms I-VIII, located in the southern region of the Amazon.

SUPPLEMENTARY REFERENCES

(1) Santos, H. G.; Jacomine, P. K. T.; Anjos, L. H. C.; Oliveira, V. Á.; Lumbreras, J. F.; Coelho, M. R.; Almeida, J. A.; Araújo-Filho, J. C.; Oliveira, J. B.; Cunha, T. J. F. Sistema Brasileiro de Classificação de Solos – SiBCS, 5th ed.; Embrapa: Brasília, DF, 2018.

(2) IOM - Institute of Medicine. Dietary Reference Intakes for Vitamin A, Vitamin K, Arsenic, Boron, Chromium, Copper, Iodine, Iron, Manganese, Molybdenum, Nickel, Silicon, Vanadium, and Zinc; National Academies Press: Washington, D.C., 2001. https://doi.org/10.17226/10026.

(3) Schrenk, D.; Bignami, M.; Bodin, L.; Chipman, J. K.; del Mazo, J.; Grasl-Kraupp, B.; Hogstrand, C.; Hoogenboom, L. (Ron); Leblanc, J.; Nebbia, C. S.; Ntzani, E.; Petersen, A.; Sand, S.; Schwerdtle, T.; Vleminckx, C.; Wallace, H.; Guérin, T.; Massanyi, P.; Van Loveren, H.; Baert, K.; Gergelova, P.; Nielsen, E. Update of the Risk Assessment of Nickel in Food and Drinking Water. EFSA J. 2020, 18 (11). https://doi.org/10.2903/j.efsa.2020.6268.

(4) FAO/WHO - Food and Agriculture Organization of the United Nation/World Health Organization. Evaluation of Certain Food Additives and Contaminants: Seventy-Third Report of the Joint FAO/WHO Expert Committee on Food Additives, WHO techni.; World Health Organization, 2011.

(5) FAO/WHO - Food and Agriculture Organization of the United Nation/World Health Organization. General Standard for Contaminants and Toxins in Food and Feed - CXS 193-1995; Codex Alimentarius, International Food Standards, 2020.

(6) Alexander, J.; Benford, D.; Cockburn, A.; Cravedi, J.-P.; Dogliotti, E.; Domenico, A. Di; Férnandez-Cruz, M. L.; Fürst, P.; Fink-Gremmels, J.; Galli, C. L.; Grandjean, P.; Gzyl, J.; Heinemeyer, G.; Johansson, N.; Mutti, A.; Schlatter, J.; Leeuwen, R. van; Peteghem, C. Van; Verger, P. Statement on Tolerable Weekly Intake for Cadmium. EFSA J. 2011, 9 (2). https://doi.org/10.2903/j.efsa.2011.1975.

(7) NASEM - National Academies of Sciences Engineering and Medicine. Nutrient Requirements of Beef Cattle, 8th Revised Edition, 8th ed.; National Academies Press: Washington, D.C., 2016. https://doi.org/10.17226/19014.

(8) Benedeti, P. D. B.; Silva, F. A. de S.; Saraiva, D. T.; Lopes, S. A.; Silva, L. F. C. e; Zanetti, D.; Valadares-Filho, S. de C. Tables of Nutritional Requirements for Beef Cattle. In Nutrient Requirements of Zebu and Crossbred Cattle - BR-CORTE; Editora Scienza, 2023; pp 417–466. https://doi.org/10.26626/978-85-8179-194-4.2023.C017.p.417-466.

(9) Ribeiro, A. C.; Guimarães, P. T. G.; Venegas, V. H. A. Recomendações Para o Uso de Corretivos e Fertilizantes Em Minas Gerais - 5a Aproximação; Comissão de Fertilidade do Solo do Estado de Minas Gerais: Viçosa - MG, 1999.
